# Compilation of the Antimicrobial Compounds Produced by *Burkholderia* Sensu Stricto

**DOI:** 10.3390/molecules28041646

**Published:** 2023-02-08

**Authors:** Mariana Rodríguez-Cisneros, Leslie Mariana Morales-Ruíz, Anuar Salazar-Gómez, Fernando Uriel Rojas-Rojas, Paulina Estrada-de los Santos

**Affiliations:** 1 Departamento de Microbiología, Escuela Nacional de Ciencias Biológicas, Instituto Politécnico Nacional, Prol. de Carpio y Plan de Ayala S/N Col. Santo Tomás Alc. Miguel Hidalgo, Ciudad de México 11340, Mexico; 2 Escuela Nacional de Estudios Superiores Unidad León, Universidad Nacional Autónoma de México (ENES-León UNAM), Blvd. UNAM 2011, León, Guanajuato 37684, Mexico; 3 Laboratorio de Ciencias AgroGenómicas, Escuela Nacional de Estudios Superiores Unidad León, Universidad Nacional Autónoma de México (ENES-León UNAM), Blvd. UNAM 2011, León, Guanajuato 37684, Mexico; 4 Laboratorio Nacional PlanTECC, Escuela Nacional de Estudios Superiores Unidad León, Universidad Nacional Autónoma de México (ENES-León UNAM), Blvd. UNAM 2011, León, Guanajuato 37684, Mexico

**Keywords:** *Burkholderia* sensu stricto, antimicrobials, non-ribosomal peptides

## Abstract

Due to the increase in multidrug-resistant microorganisms, the investigation of novel or more efficient antimicrobial compounds is essential. The World Health Organization issued a list of priority multidrug-resistant bacteria whose eradication will require new antibiotics. Among them, *Acinetobacter baumannii, Pseudomonas aeruginosa*, and Enterobacteriaceae are in the “critical” (most urgent) category. As a result, major investigations are ongoing worldwide to discover new antimicrobial compounds. *Burkholderia*, specifically *Burkholderia* sensu stricto, is recognized as an antimicrobial-producing group of species. Highly dissimilar compounds are among the molecules produced by this genus, such as those that are unique to a particular strain (like compound CF66I produced by *Burkholderia cepacia* CF-66) or antimicrobials found in a number of species, e.g., phenazines or ornibactins. The compounds produced by *Burkholderia* include N-containing heterocycles, volatile organic compounds, polyenes, polyynes, siderophores, macrolides, bacteriocins, quinolones, and other not classified antimicrobials. Some of them might be candidates not only for antimicrobials for both bacteria and fungi, but also as anticancer or antitumor agents. Therefore, in this review, the wide range of antimicrobial compounds produced by *Burkholderia* is explored, focusing especially on those compounds that were tested in vitro for antimicrobial activity. In addition, information was gathered regarding novel compounds discovered by genome-guided approaches.

## 1. Introduction

*Burkholderia* sensu lato comprises more than 100 species, which were gradually discovered during 30 years of research. In recent years, using comparative genomics, this large group was divided into seven genera, namely *Burkholderia* sensu stricto (s.s.), *Paraburkholderia*, *Caballeronia*, *Robbsia*, *Mycetohabitans*, *Trinickia*, and *Pararobbsia* [1,2,3,4,5]. The species contained in these genera thrive in soil, water, rhizosphere, plant nodules, fungi, and in animal and human infections. *Burkholderia* s.s. is formed by three groups of species: (a) the *Burkholderia pseudomallei* group (composed of 8 species), (b) the *Burkholderia* species that are mostly plant pathogenic bacteria (containing 4 species), and (c) the *Burkholderia cepacia* complex (Bcc) (composed of 25 species). The *B. pseudomallei* group is of worldwide importance because the species *B. pseudomallei* and *Burkholderia* mallei cause the mortal (if not treated) melioidosis diseases in humans and animals, and glanders, specifically in equines, respectively [6,7]. Recently, “*Burkholderia mayonis*” and “*Burkholderia savannae*” were described within the *B. pseudomallei* group [8]. Although some species from the Bcc are plant pathogens, there is a small group where *Burkholderia plantarii*, *Burkholderia gladioli*, and *Burkholderia glumae* are included; however, they are not part of the Bcc. Recently, “*Burkholderia perseverans*” was added to this group; this species produces volatile compounds that inhibit plant pathogens but has not been described as a pathogen per se [9]. The Bcc species are best known as opportunistic pathogens, mainly in cystic fibrosis (CF) and immunocompromised patients [10]. Within the Bcc, the last species described was *Burkholderia orbicola* [11]. Another important feature within the Bcc is their resistance to many antibiotics [12], which especially endangers the lives of CF and immunocompromised patients.

Bcc is also known for their phenotypic and genotypic diversity [13], which includes features/functions for biotechnological uses. This functional ability has been shown through biopesticidal activity in the rhizosphere [14] and by the bioremediation of xenobiotics compounds [15,16]. The Bcc are also able to produce a large array of compounds involved in the inhibition of pathogenic bacteria, fungi, and yeasts, which is important for tackling multidrug-resistant microorganisms [17]. Interestingly, the *B. pseudomallei* group encodes the largest capacity for secondary metabolite biosynthesis (>11% of their genomes) [18]. Moreover, the Bcc account for significant antibiotic biosynthetic capacity, e.g., *Burkholderia ambifaria* involves 9% of its genome in secondary metabolism, and *B. gladioli* and *B. glumae* dedicate 10% or more of their genome to antibiotic biosynthesis. Therefore, this review aims to enumerate in detail all antimicrobial compounds produced by *Burkholderia* s.s., detailing activities demonstrated in vitro and reviewing the novel compounds discovered by genome-guided approaches. The compounds are grouped and discussed according to common chemical features and shown as a list in Supplementary Table S1 with the numbers given in bold.

## 2. N-Containing Heterocycles

**Figure 1:**
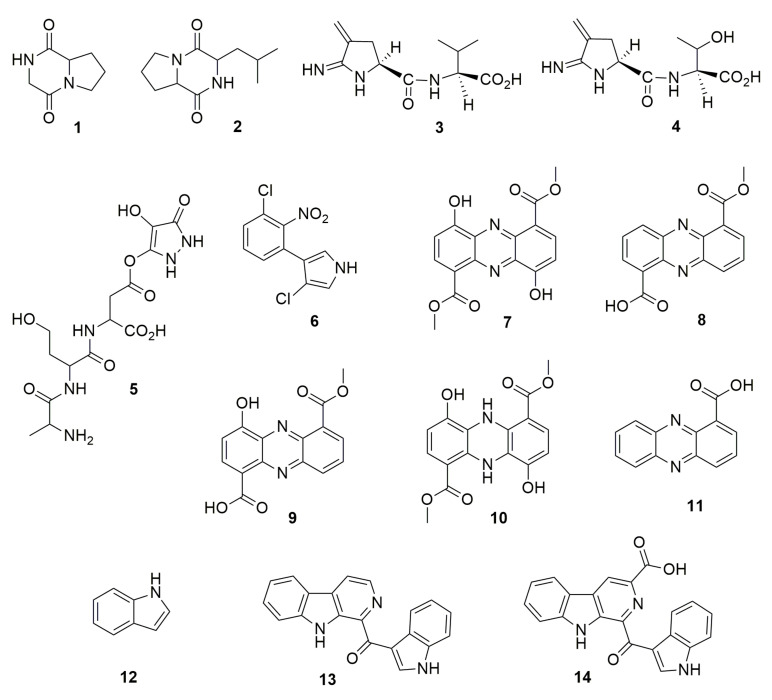
N-containing heterocycles. (**1**) pyrrolo (1,2-a) pyrazine-1,4-dione, hexahydro. (**2**) pyrrolo (1,2-a) pyrazine-1,4-dione, hexahydro-3(2-methyl-propyl). (**3**) 2-imino-3-methylene-5-L(carboxy-L-valyl)-pyrrolidine. (**4**) 2-imino-3-methylene-5-L(carboxy-L-threoninyl)-pyrrolidine. (**5**) 3-[L-alanyl-L-*homoserinyl*-L-aspartyl-b-carboxy]-4-hydroxy-5-oxopyrazole. (**6**) pyrrolnitrin, 3-chloro-4-(2-nitro-3-chlorophenyl)pyrrole. (**7**) phenazine, 4,9-dihydroxyphenazine-1,6-dicarboxylic acid dimethyl ester. (**8**) phencomycin. (**9**) 4-hydroxyphencomycin. (**10**) 5,10-dihydro-4,9-dihydroxyphencomycin methyl ester. (**11**) phenazine-1-carboxilic acid. (**12**) índole. (**13**) pityriacitrin. (**14**) pityriacitrin B.

The analogs of nitrogen-based heterocycles occupy an exclusive position as a valuable source of therapeutic agents in medicinal chemistry [19]. Many of these compounds are volatile organic compounds (VOCs) or volatile nitrogen compounds [20]. **Pyrazine**-derived compounds (VOC) produced by *Burkholderia seminalis* JRBHU6 have been named PPDH and identified as (pyrrolo (1,2-a) pyrazine-1,4-dione, hexahydro) (**1**) and PPDHMP identified as C_11_H_18_N_2_O_2_ (pyrrolo (1,2-a) pyrazine-1,4-dione, hexahydro-3(2-methyl-propyl)) (**2**) [21]. The pyrrolo [1,2-a] pyrazine core occurs in nature and is frequently used in drug design. Pyrrole has therapeutic significance as an anticancer, antimicrobial, and antiviral agent [22]. Compounds **1** and **2** produced by *B. seminalis* JRBHU6 inhibit the fungi genera *Fusarium, Aspergillus, Microsporum, Trichophyton*, and *Trichoderma*, and the bacterial genera *Staphyloccous*, *Pseudomonas*, *Escherichia*, *Shigella*, and *Klebsiella* [21]. Molecular docking with bioactive compounds **1** and **2** was carried out to identify protein targets. According to the analysis, **2** showed full fitness to human proteins cell division protein kinase 7 and mitogen-activated protein kinase 8, suggesting a putative role in the inhibition of protein kinase activity in sensitive microorganisms. Good binding affinity and full fitness were also found with bacterial proteins such as choloylglycine hydrolase, camphor t-monooxygenase, chitinase B, and tyrosine phenol-lyase, while **1** showed good strong full fitness only with chitinase B. Other N-containing antimicrobial compounds are **iminopyrrolidines** produced by *B. plantarii* 9424. These compounds are 2-imino-3-methylene-5-L(carboxy-L-valyl)-pyrrolidine (**3**) and 2-imino-3-methylene-5-L(carboxy-L-threoninyl)-pyrrolidine (**4**). These are amino acid conjugates and have high in vitro inhibitory activity against the bacteria *Erwinia amylovora*, a pathogen causing fire blight disease in apple and pear trees [23]. A **pyrazole molecule** that consisted of a substituted pyrazole, linked to the aspartate-b-carboxyl of the tripeptide L-alanyl-L-*homoserinyl*-L-aspartate, resulted in a deduced structure 3-[L-alanyl-L-*homoserinyl*-L-aspartyl-b-carboxy]-4-hydroxy-5-oxopyrazole (**5**). This compound was produced by *B. glumae* and was found to inhibit bacterial pathogens such as different species of *Erwinia*, *Pectobacterium*, *Pseudomonas*, and *Xanthomonas* [24]. **Pyrrolnitrin** (**6**), 3-chloro-4-(2-nitro-3-chlorophenyl)pyrrole, is a microbial halometabolite (containing a halogen moiety) with a large antimicrobial significance in agricultural, pharmaceutical, and industrial implications [25]. This compound is produced by rhizospheric fluorescent and non-fluorescent pseudomonads, *Serratia* and *Burkholderia*. Pyrrolnitrin was first discovered in *Pseudomonas* (now *Burkholderia*) *pyrrocinia* in 1960 [26,27]; other species such as *Burkholderia cepacia* and *Burkholderia ambifaria* are able to synthesize it as well [28,29,30,31]. A number of phytopathogens are inhibited by **6**, e.g., Penicillium, Phytophthora, *Fusarium*, *Rhizoctonia*, *Colletotrichum*, and *Sclerotinia*, yeast such as *Candida*, *Hansenula*, and *Saccharomyces*, and bacteria such as *Bacillus* and *Streptomyces*. Interestingly, the production of pyrrolnitrin was induced when chloramphenicol was added to the culture medium of *B. ambifaria* AMMD^T^ [31]. **Phenazines** are a large group of nitrogen-containing heterocycles with diverse chemical structures and pharmacological activity such as antimicrobial, antiparasitic, neuroprotective, insecticidal, anti-inflammatory, and anticancer [32]. There are more than 100 phenazine derivatives produced by bacteria and archaea. *Burkholderia cepacia* 5.5B produces the phenazine 4,9-dihydroxyphenazine-1,6-dicarboxylic acid dimethyl ester (**7**), which inhibits *Rhizoctonia solani* [33]. The production of phenazines in *B. lata* was strongly affected by the growth conditions, the best production being observed in culture grown in King’s B medium [34]. Moreover, the involvement of phenazine in the formation of biofilm by *B. lata* was analyzed using a phenazine-overproducing strain, a phenazine-deficient mutant, and the wild type of the strain. The results showed that both the wild type and the overproducing strain formed thicker biofilms and attached more quickly than the mutant, suggesting a role of phenazine in biofilm formation by *B. lata* and, therefore, a role in the pathogenicity of this member of Bcc. *Burkholderia glumae* 411gr-6 was found to synthesize **phencomycin** (**8**) (a phenazine with two substituents, a carboxyl and a carbomethoxy group) and two new derivatives, 4-hydroxyphencomycin (**9**) and 5,10-dihydro-4,9-dihydroxyphencomycin methyl ester (**10**) [35]. The three compounds inhibit several plant pathogenic fungi, yeasts, and bacteria. *Burkholderia* sp. HQB-1, closely related to *Burkholderia stagnalis*, produces PCA, **phenazine-1-carboxilic acid** (**11**), which has been proposed to protect banana against *Fusarium* oxysporum wilt. Compound **11** produced by strain HQB-1 also inhibits the genera *Colletotrichum*, Botrytis, and *Curvularia* [36]. **Indole** compounds and derivatives are N-containing heterocycles; among this kind of compounds the VOC indole (**12**) produced by *Burkholderia cenocepacia* ETR-B22 inhibited the fungi *Alternaria, Aspergillus, Bipolaris, Bacillus, Fusarium, Helminthosporium, Mycosphaerella, Magnaporthe, Phyllosticta*, and *Rhizoctonia* [37]. The **pityriacitrin** (**13**), a b-carboline alkaloid with an indole ring attached with a carbonyl group on C-1 position and the derivative **pityriacitrin B** (**14**) isolated and identified in *Burkholderia* sp. NBF227, was tested for cytotoxic activity against cancer cell lines, but chemically synthesized derivatives from the previous compounds were more effective [38]. Other synthesized pityriacitrin derivatives from *Burkholderia* sp. NBF227 were investigated for antifungal activity [39]. The fungicidal activity was tested with four taxonomically different plant pathogens (oomycetes, ascomycetes, deuteromycetes, and basidiomycetes), showing that pityriacitrin displayed broad-spectrum antifungal activity and protected pepper leaves and grapefruits against infection by *P. capsici* and *B. cinerea*, respectively. Some N-containing heterocycles are shown in Figure 1.

## 3. Volatile Organic Compounds

**Figure 2:**
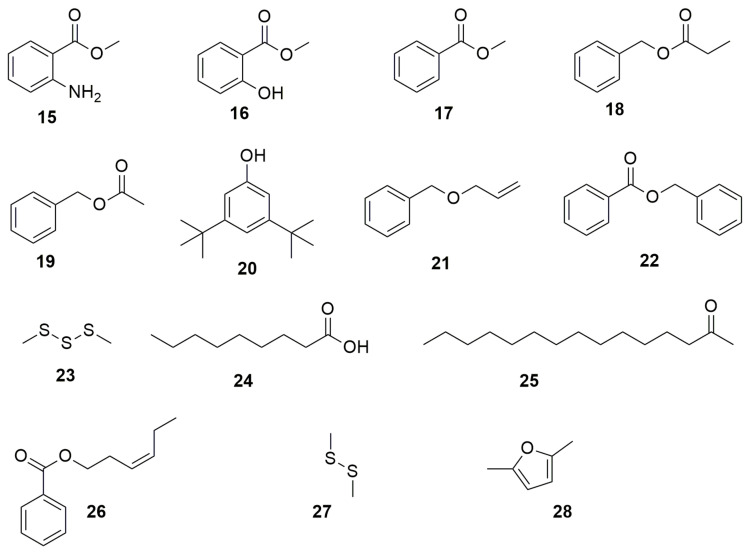
Volatile organic compounds. (**15**) methyl anthranilate. (**16**) methyl salicylate. (**17**) methyl benzoate. (**18**) benzyl propionate. (**19**) benzyl acetate. (**20**) 3,5-Di-tert-butylphenol. (**21**) allyl benzyl ether. (**22**) benzyl benzoate. (**23**) dimethyl trisulfide. (**24**) nonaoic acid. (**25**) 2-pentadecanone. (**26**) 3-hexen-1-ol, benzoate, (Z)-. (**27**) dimethyl disulfoxide. (**28**) 2,5-dimethylfuran.

Besides the VOCs mentioned in the N-containing heterocycles section, *B. cenocepacia* ETR-B22 also synthetizes other VOCs that lack nitrogen in their ring structure (Figure 2). These compounds are the benzyl derivatives **methyl anthranilate** (**15**), **methyl salicylate** (**16**), **methyl benzoate** (**17**), **benzyl propionate** (**18**), **benzyl acetate** (**19**), **3,5-Di-tert-butylphenol** (**20**), **allyl benzyl ether** (**21**), and **benzyl benzoate** (**22**) (Figure 2), which inhibit an important number of fungal plant pathogens [37]. The VOCs **dimethyl trisulfide** (**23**), **nonanoic acid** (**24**), **2-pentadecanone** (**25**), and 3**-hexen-1-ol, benzoate, (Z)-** (**26**) produced by the strain ETR-B22 also have antifungal activity. *Burkholderia gladioli* strain BBB-01, isolated from rice shoots, emits the VOCs **dimethyl disulfide** (**27**) and **2,5-dimethylfuran** (**28**) with inhibitory activity against the phytopathogenic fungi *M. oryzae*, *Gibberella fujikuroi*, *Sarocladium oryzae*, *Phellinus noxius*, and *Colletotrichum fructicola* and human pathogen *C. albicans* [40].

## 4. Polyenes

**Figure 3:**
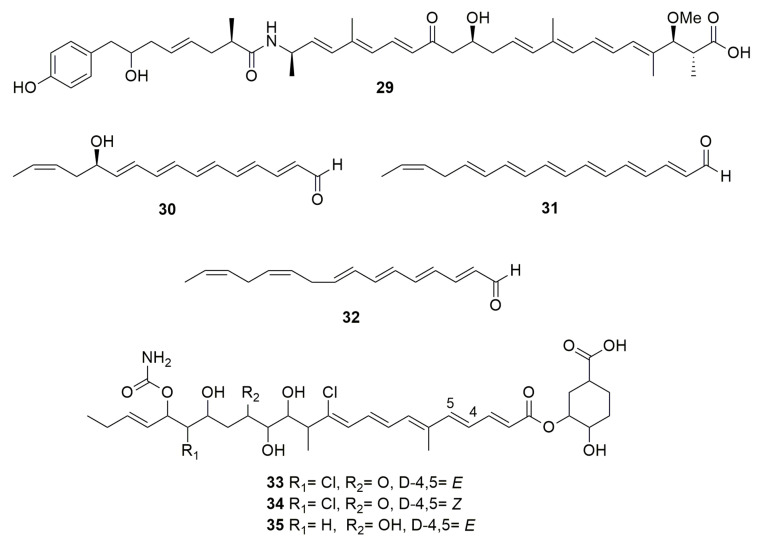
Polyenes. (**29**) thailandamide A. (**30**) thailandene A. (**31**) thailandene B. (**32**) thailandene C. (**33**) enacyloxin IIa. (**34**) iso-enacyloxin IIa. (**35**) enacyloxin IIIa.

Polyenes are poly-unsaturated organic compounds that contain at least three alternating double and single carbon–carbon bonds. Hunter and Manter [41] reported the isolation and purification of a compound with oxidizing and antibiotic properties from *B. cenocepacia* P525. The structure of this compound has not been reported but the preliminary chemical study showed that the compound could be a **polyene** with six conjugated double bonds and bacteriostatic activity against Enterobacter soli and Enterobacter aerogenes. *Burkholderia thailandensis* is a close relative of *B. pseudomallei* and therefore used as a model to study *B. pseudomallei* pathogenicity and biosynthetic pathways because *B. thailandensis* is not a pathogen. This species produces the polyene polyketide **thailandamide A** (**29**) (Figure 3) inhibiting notably bacteria such as *Bacillus subtilis*, *S. aureus*, and *Neisseria gonorrhoeae* [42]. Genetic analysis showed that **29** inhibits acetyl-CoA carboxylase (ACC), an essential enzyme responsible for the first step in fatty acid biosynthesis. Moreover, *B. thailandensis* synthetizes **thailandenes A** (**30**)**, B** (**31**) and **C** (**32**) (Figure 3), which are linear formylated or acidic polyenes containing a combination of cis and trans double bonds [43]. Compounds **30** and **31** exhibited potent antimicrobial activity against *S. aureus* and *S. cerevisiae*. A polyketide (PK) **enacyloxin IIa** (**33**) (Figure 3) and its stereoisomer, designated **iso-enacyloxin IIa** (**34),** produced by *B. ambifaria* AMMD^T^, has activity against *Burkholderia multivorans*, *Burkholderia dolosa*, and *Acinetobacter baumannii* [44]. Expression analysis showed that enzymes-encoding genes for enacyloxin biosynthesis were among the most highly upregulated when strain AMMD^T^ was grown to stationary phase on glycerol. Moreover, enacyloxin targets protein biosynthesis by inhibition of the ribosomal elongation factor Tu [45]. *Burkholderia gladioli* pv. cocovenenans ATCC 33664^T^ produces **33** and **enacyloxin IIIa** (**35**), and both were found to display equally potent activity against *Escherichia coli* and *P. aeruginosa* [46]. Moreover, traditionally used in food fermentations (tempe and sufu), Rhizopus microspores is accompanied by *B. gladioli* pv. cocovenenans. Thus, a coculture of both microorganisms showed that enacyloxins were found in high titers, with an increased production of the lethal toxin bongkrekic acid, showing the significance to food safety of this common microbial co-existence.

## 5. Polyynes

**Figure 4:**
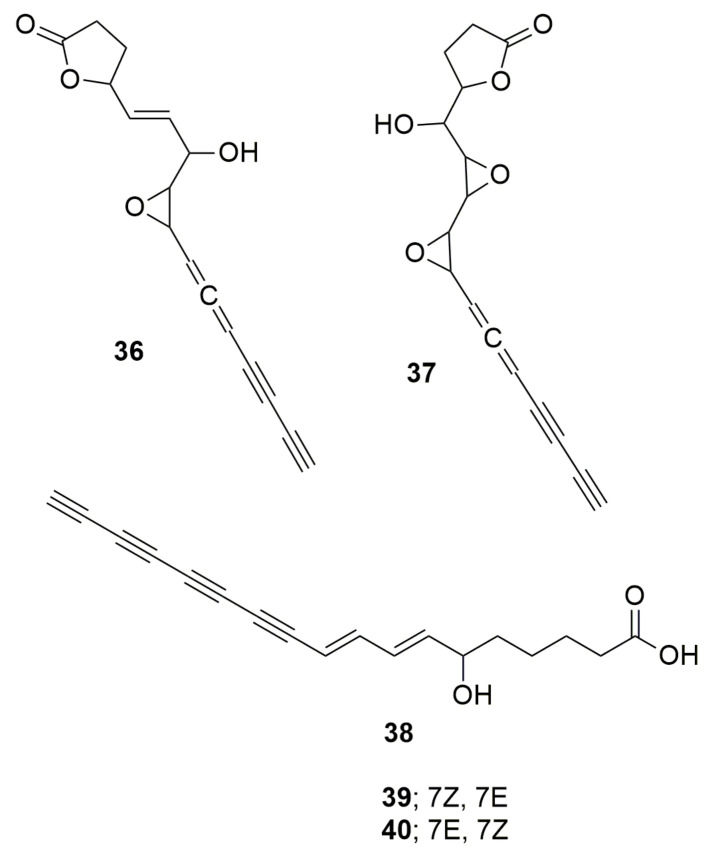
Polyynes. (**36**) Cepacin. (**37**) Cepacin B. (**38**) Caryoynecin A. (**39**) Caryoynecin B. (**40**) Caryoynencin C.

Polyynes are organic compounds with alternating single and triple bonds, a series of consecutive alkynes. **Cepacin A** (**36**) and **cepacin B** (**37**) (Figure 4) are two acetylenic antibiotics produced by *B. cepacia* SC 11,783 with a strong activity against staphylococci [47] (Parker et al. 1984). *Burkholderia ambifaria* BCC0191 also synthetizes the metabolite cepacin A, which mediates protection of germinating crops against *Pythium* damping-off disease [48]. The activity was demonstrated when no biological control was observed with the inoculation of a cepacin mutant of strain BCC0191. *Burkholderia caryophylli*, a plant pathogen, produces the triple-bond compounds **caryoynecin A** (**38**), **B** (**39**), **and C** (**40**) (Figure 4). Although they are unstable, they can inhibit *E. coli*, *K. pneumoniae*, and *S. aureus* [49]. Caryoynecin analogues synthesized chemically were found more stable and demonstrated activity against *S. aureus, B. subtilis, Enterococcus faecalis, E. coli, Salmonella enteritidis, K. pneumoniae, Serratia marcescens, Proteus vulgaris, Shigella flexneri, Enterobacter cloacae, P. aeruginosa, T. mentagrophytes, Trichophyton interdigitale*, and *Trichophyton rubrum* [50]. *Burkholderia gladioli* also produces caryoynencin, which has activity against Purpureocillium lilacinum and has a role in the transition of the plant pathogen to an insect-defensive mutualism [51].

## 6. Siderophores

**Figure 5:**
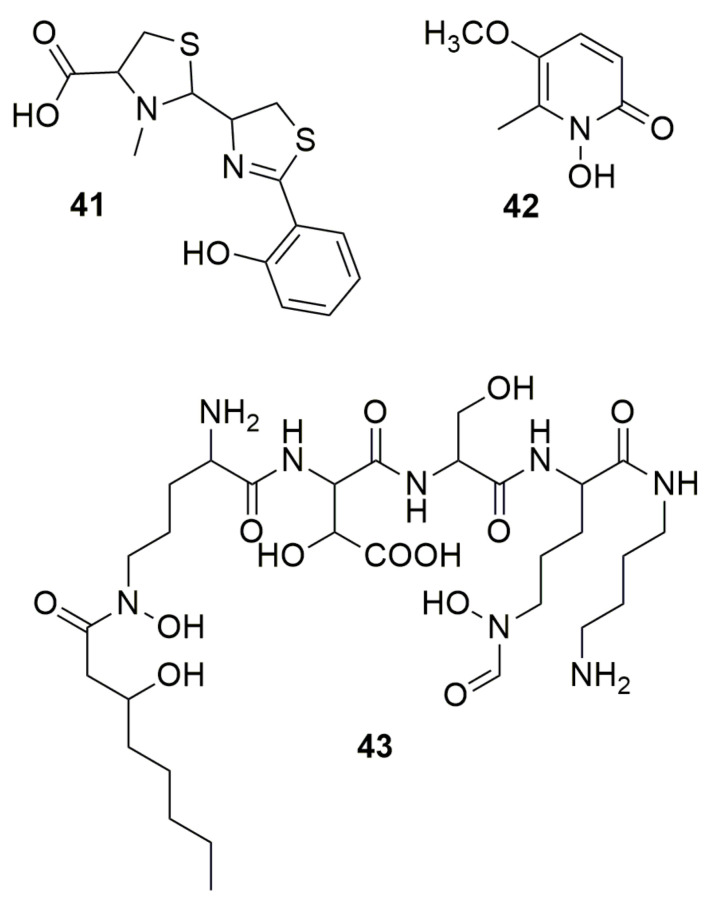
Siderophores. (**41**) Pyochelin. (**42**) Cepabactin. (**43**) Ornibactin.

Siderophores are low-molecular-weight organic compounds with high affinity to chelate iron (Fe). These compounds are produced by microorganisms and higher plants [52]. The typical siderophores ligands are cathecholate, *a*-hydrocycarboxylate, hydroxyphenyloxazolone, hydroxamate, *a*-aminocarboxylate, and a-hydroxyimidazole. Many bacterial siderophores are synthesized through **non-ribosomal peptide synthetases** (**NRPS**). NRPS are a large family of biosynthetic enzymes that generate relevant natural compounds from amino acid precursors [53,54]. NRPSs are frequently categorized as type I and II [55]. Type I NRPSs are large modular complexes containing all the enzymes necessary to generate a peptide product in an assembly line fashion analogous to type I fatty acid synthases (FASs) and polyketide synthases (PKSs). Type II NRPS proteins are commonly standalone enzymes or didomains that coordinate to form unique amino acid derivatives. Unlike type II FAS and PKS, the type II NRPS proteins are linear, noniterative pathways that contain specialized tailoring enzymes and combine with other pathways to generate a final product [55]. **Pyochelin** (**41**) (Figure 5), a non-ribosomal peptide (NRP) purified from *P. aeruginosa* PAO1, was first found to display antibiotic activity against *S. aureus* and moderately against several species of *Xanthomonas* [56]. Pyochelin produced by “*Burkholderia paludis*”, a non-validated species within the Bcc, inhibits three multidrug-resistant *E. faecalis* and four *S. aureus* strains but was not able to inhibit *Bacillus subtilis* ATCC 8188, *Bacillus cereus* ATCC 14579, *Aeromonas hydrophila* ATCC 49140, *E. coli* ATCC 25922, *Klebsiella pneumoniae* ATCC 10031, *Proteus mirabilis* ATCC 49140, *P. vulgaris* IMR, *P. aeruginosa* ATCC 10145 and ATCC BAA-47, *Salmonella Typhimurium* ATCC 14028, or *Shigella flexneri* ATCC 12022 [57]. This compound enhanced the production of intracellular reactive oxygen species (ROS), leading to cell death by disrupting the integrity of the bacterial membrane [58]. Pyochelin synthesized by *B. seminalis* TC3.4.2R3 inhibits *F. oxysporum*, which was demonstrated when a cepacin mutant was unable to inhibit the fungi [59]. **Cepabactin** (**42**) (Figure 5) is a 1-hydroxy-5-methoxy-6-methyl-2(1H)-pyridinone, a cyclic hydroxamate but also a heterocyclic analogue of catechol [60]. This compound produced by *B. cepacia* ATCC 25416^T^ has antimicrobial activity against *S. aureus*, *Staphylococcus epidermidis*, *Streptococcus faecalis*, *B. subtilis*, *Bacillus anthracis*, *E. coli*, *Salmonella Typhi*, *Salmonella Typhimurium*, *K. pneumoniae*, *P. vulgaris*, *P. mirabilis*, and *Proteus rettgeri* [60,61,62]. The production of **42** was present in only 12% of 65 *B. cepacia* strains, lower than other siderophores such as ornibactin (87%) or pyochelin (60%), showing that this siderophore is not largely produced in the species [63]. **Ornibactin** (**43**) (Figure 5), a NRP produced by most *Burkholderia* species [64], is a tetrapeptide siderophore with an l-ornithine-d-hydroxyaspartate-l-serine-l-ornithine backbone. A study with *Burkholderia* contaminans MS14, isolated from soil in Mississippi, USA, using transposon mutagenesis, resulted in two strains with insertional mutations in orbI gene (mutant MT577) and a luxR family transcriptional regulatory gene (mutant MT357) [65]. Both mutants lost bactericidal activity, relating the activity to siderophore ornibactin. This compound successfully inhibited *Xanthomonas citri* pv. malvacearum, *P. carotovorum* supsp. carotovorum, *Ralstonia solanacearum, P. syringae* pv. syringae, *E. amylovora*, *E. coli*, Clavibacter michiganensis subsp. Michiganensis, and *Bacillus* megaterium. The ornibactin mutant retained antifungal activity, showing that the antibacterial and antifungal action is independent, with the antifungal activity a result of occidiofungin. Similarly, ornibactin derivatives produced by *Burkholderia catarinensis* 89^T^ presented no activity against fungi [17]. Pyochelin and ornibactin are siderophores found in the genome of *Burkholderia orbicola* TAtl-371^T^, and this bacterium produces siderophores in culture medium [66]. A test removing iron from the culture medium showed that the bacteria was able to inhibit *Paraburkholderia phenazinium* and *Candida glabrata*, suggesting the involvement of these siderophores in antagonism.

## 7. Macrolides

**Figure 6:**
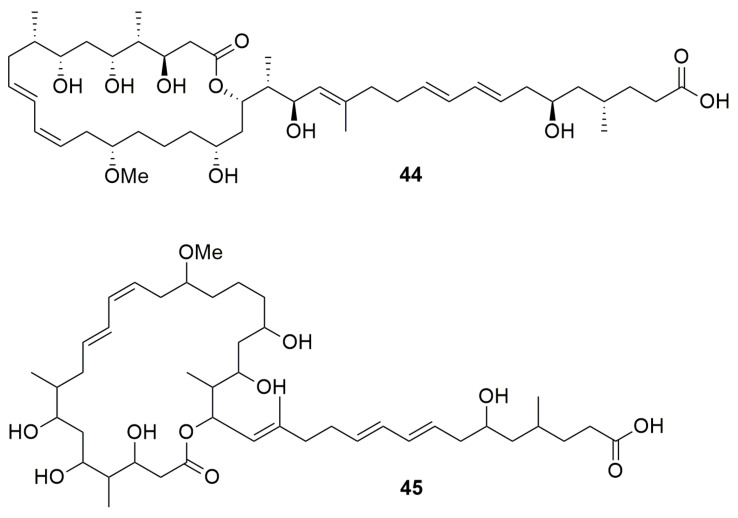
Macrolides. (**44**) Gladiolin. (**45**) Lagriene.

Macrolides are various types of hydrophobic compounds containing a macrocyclic lactone ring and various side chains/groups [67]. *Burkholderia gladioli* BCC0238 isolated from a CF patient synthesize the PK, macrolide antibiotic **gladiolin** (**44**) (Figure 6), which has a strong activity against *M. tuberculosis* H37Rv and several other *M. tuberculosis* strains, *K. pneumoniae*, *A. baumannii*, *P. aeruginosa*, *E. clocae*, *Serratia plymuthica*, “*Ralstonia mannitolilytica*”, *B. multivorans*, *E. coli*, *Enterococcus faecium*, *S. aureus*, *B. subtilis*, and *C. albicans*, and was found to exhibit low toxicity toward an ovarian cancer cell line [68]. The mode of action of gladiolin is the inhibition of the RNA polymerase. Another PK macrolide **lagriene** (**45**) (Figure 6), produced by *B. gladioli* Lv-StA, has activity against *B. thuringiensis*, *M. vaccae*, vancomycin-resistant *E. faecalis* and *S. aureus* [51].

## 8. Bacteriocins

Bacteriocins are a varied class of bactericidal peptides or proteins produced by bacteria and archaea with bactericidal activity and specific immunity mechanisms toward strains closely related to the producer bacteria [69]. There are two central differences between bacteriocins and antibiotics: bacteriocins are ribosomally synthesized but antibiotics are not, and bacteriocins have a somewhat narrow killing spectrum while antibiotics have an extensive killing range. Bacteriocins vary in size, microbial target, mode of action, release, and immunity mechanism, and can be divided into two groups, the ones produced by Gram-negative bacteria and those by Gram-positive bacteria. Gram-negative bacteriocins are further classified according to their size into three main groups, namely colicins, phage-tail-like bacteriocins, and microcins [70]. Microcins are low-molecular-weight compounds grouped into class I (<5 kDa) or class II (5–10 kDa). Class I is now designated as ribosomally synthesized and post-translationally modified peptides (RiPP). *Burkholderia cenocepacia* BC0425 synthesizes the bacteriocin **tailocin**, a phage tail-like compound, named **BceTMilo** [71]. Unlike phages, tailocin injects through the cell membrane and disrupts the proton motive force [72]. Strains belonging to Bcc are sensitive to BceTMilo, and other non-Bcc such as *B. gladioli* and *B. glumae* are also sensitive to tailocin. Lectin-like bacteriocins (**LlpAs**) contain two monocot mannose-binding lectin (MMBL) domains, a module predominantly and abundantly found in lectins from monocot plants. *Burkholderia* strains can synthesize these bacteriocins. *B. cenocepacia* AU1054 (now *B. orbicola*) [11] produces an LlpA bacteriocin that inhibits *B. ambifaria*, *Burkholderia anthina*, *B. cenocepacia*, *B. contaminans* and *Burkholderia metallica* [73]. The homologue LlpA88 from *B. orbicola* TAtl-371^T^ inhibited the same species as strain AU1054 [66]. **Burkhocins M1** and **M2**, colicin M-like bacteriocins called ColM in *E. coli*, from *B. ambifaria* MEX-5 and AMMD^T^ were produced recombinantly, showing antagonistic activity against a number of Bcc strains [74]. Three strains from *Burkholderia ubonensis* inhibited *B. pseudomallei*; the antagonism from a representative strain (A21) was characterized, and a pepsin-sensitive moiety consistent with a bacteriocin-like compound was found, suggesting the antagonism is due to the production of a bacteriocin or bacteriocin-like inhibitory substance (**BLIS**) [75]. **Lasso peptides** are a structurally unique class of bioactive peptides characterized by a knotted arrangement where the C-terminus threads through an N-terminal macrolactam ring [76]. Lasso peptides are divided depending on the presence (class I) or absence (class II) of four conserved cysteine residues involved in the formation of two intramolecular disulfide bonds [77]. *Burkholderia thailandensis* produces the lasso peptide **capistruin**, a 19-amino-acid class II lasso peptide comprising an isopeptide bond between Gly1 and Asp9 resulting in a nine-residue macrolactam ring [78], which exhibits antimicrobial activity against *Burkholderia* (now *Paraburkholderia*) *caledonica*, *E. coli*, and *P. aeruginosa* [76]. **Ubonodin**, another lasso peptide produced by *B. ubonensis* MSMB2207, was heterologous expressed in *E. coli* BL21, displaying inhibition of *B. cepacia*, *B. multivorans* and *B. mallei*; it has a weak effect against *B. thailandensis* and had no effect on *B. gladioli* and *B. pseudomallei* [79]. This compound inhibits RNA polymerase in vitro and the narrow effect might allow therapeutic usage.

## 9. Quinolones

**Figure 7:**
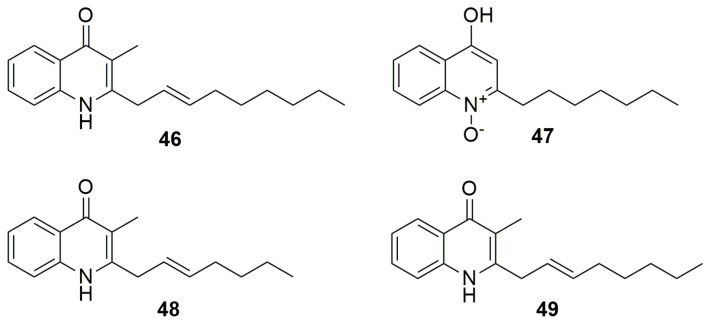
Quinolones. (**46**) HMNQ (4-hydroxy-3-methyl-2-(2-nonenyl)-quinoline). (**47**) HQNO (2-heptyl-4(1H)-quinolone N-oxide). (**48**) 2-(2-heptenyl)-3-methyl-4-quinolinol (C7Δ2). (**49**) Burkholone.

Quinolones were discovered as a by-product in the search for improved synthesis of the anti-malarial chloroquine; thus, they are fully synthetic molecules [80]. Today, it is known that molecules in the quinolone family are also present as natural products of plants and bacteria, although their potency has been tested only at the experimental level. The basic structure is a 3-carboxyquinolone and the first quinolone described was nalidixic acid. *Burkholderia thailandensis* contains a biosynthetic gene cluster (BGCs), which is a quorum-sensing-regulated *hmq* cluster that produces a diverse set of hydroxyalkylquinolines (HAQs). These compounds exist mainly in the 4(1H) quinolone type at neutral pH and are known as bioactive metabolites [81]. Two HAQ analogues, **HMNQ** (4-hydroxy-3-methyl-2-(2-nonenyl)-quinoline) (**46**) and **HQNO** (2-heptyl-4(1H)-quinolone N-oxide) (**47**) (Figure 7), synthesized by *B. thailandensis* E264^T^, when challenged with antibiotics inhibit *B. subtilis* 168 but display weak activity against *E. coli* K12 [82]. It was found, as well, that both quinolones act synergistically to inhibit bacterial growth. Moreover, *B. thailandensis* produces **46** and rhamnolipids in outer membrane vesicles (OMV), which have antimicrobial and antibiofilm properties against methyl-resistant *S. aureus* [83]. Bacterial OMVs contain proteins, lipids, polysaccharides, and small molecules and serve numerous and versatile roles in intra- and interspecies interactions. *Burkholderia cepacia* RB425, isolated from lettuce root, makes the quinolone antibiotics **2-(2-heptenyl)-3-methyl-4-quinolinol** (**48**) and (**46**) (Figure 7) with high activity against fungal pathogen Verticillium dahlia, moderate inhibition of *Pyricularia oryzae* and *Cochliobolus myyabeanus*, and weak growth inhibition of *R. solani*, *F. oxysporum*, and *Gaeumannomyces graminis* [84]. A range of hydroxy-methyl-alkylquinolines (**HMAQ**) produced by *B. cepacia* PC-II antagonizes *P. capsici*, which is responsible for Phytophthora blight in red peppers and many vegetables; in particular, **48** was the most potent against the oomycetes *P. capsica* and *Pythium* ultimum and the fungi *F. oxysporum* and *R. solanc* [85]. *Burkholderia* sp. QN15488 produces **burkholone** (**49**) (Figure 7), a (E)-3-methyl-2-(octenyl)-4-quinolone, this compound induces cell death in 32D/GR15 cells in IGF-I-containing medium [86]. Insulin-like growth factors (IGFs) play a key role in human cancer progression and IGF signals through the IGF-1 receptor are known to be significant for tumor cell growth and survival [87].

## 10. Other NPR-PK Compounds

**Figure 8:**
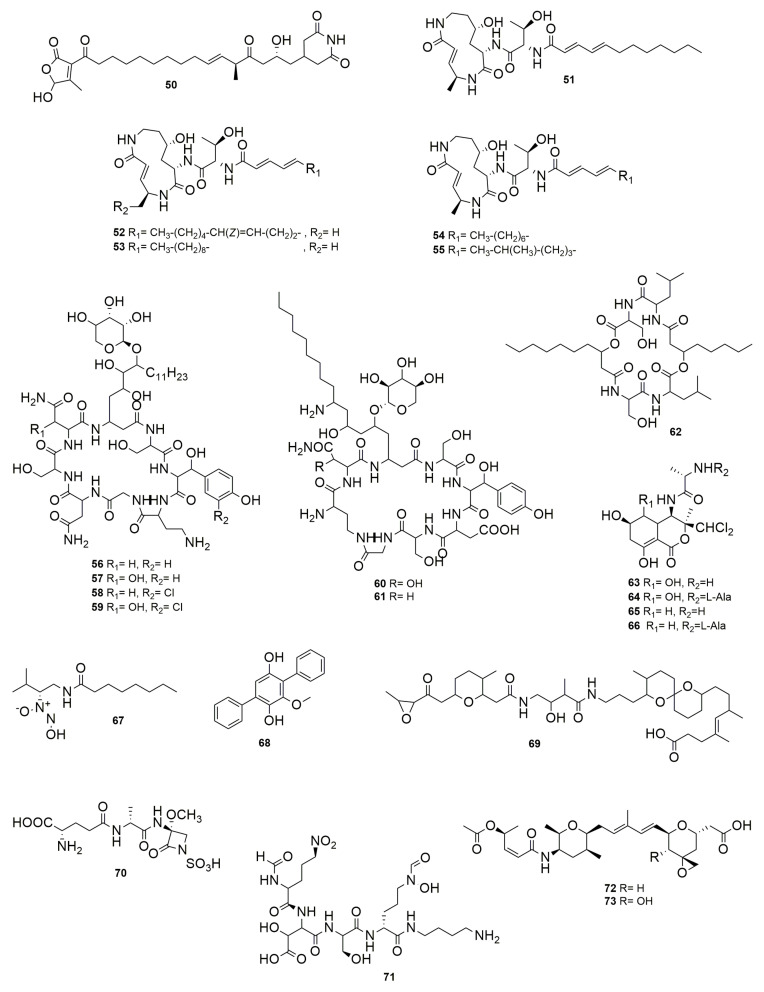
Other NPR-PK compounds. (**50**) Gladiofungin A. (**51**) Glidobactin A. (**52**) Glidobactin B. (**53**) Glidobactn C. (**54**) Cepafungin I, II (**55**) Cepafungin III. (**56**–**59**) Occidiofungins A–D. (**60**) Cepacidine A_1_. (**61**) Cepacidine A_2_. (**62**) Icosalide A1. (**63**–**66**) Bactobolins A–D. (**67**) Fragin. (**68**) BTH-II0204-207:A. (**69**) Lagriamide. (**70**) Isosulfazecin (iSZ). (**71**) Malleonitrone. (**72**–**73**) Spliceostatins.

**Gladiofungin A** (**50**) (Figure 8) is a novel antifungal PK that is highly unusual because it harbors a butanolide moiety. This compound is produced by the insect-associated bacteria *B. gladioli* HKI0739, which displays activity against *Penicillium notatum*, *Sprobolomyces salmonicolor*, and *P. lilacinum* [88]. The strains BCC0238 and BCC1622, belonging to *B. gladioli*, produce a PK antibiotic named **gladiostatin**, which has the same structure as (**50**) [89]. This molecule has promising activity against several cancer cell lines such as ovarian, pancreatic, and colon cancer, and inhibits tumor cell migration. Moreover, it was found to be inactive against a lung cell line, which indicates that it may exhibit some selectivity. Gladiostatin contains an unusual 2-acyl-4-hydroxy-3-methylbutenolide in addition to the glutarimide pharmacophore that also inhibits *S. cerevisiae*. **Glidobactins A** (**51**), **B** (**52**), and **C** (**53**) have a common cyclized tripeptide nucleus composed of L-threonine, 4(S)-amino-2(E)-pentoic acid, and erythro-4hydroxy-L-lysine but differ from each other in the unsaturated fatty acid moiety attached to the peptide [90]. These compounds were isolated from strain K481-B101, whose 16S rRNA sequence (Accession No. AM410613) analyzed in the EzBioCloud server (https://www.ezbiocloud.net/, accessed on 1 June 2022) was identified as *Schlegelella brevitalea*, a member of the Burkholderiales order and Comamonadaceae family. These glidobactins compounds have antifungal and antitumor activity [91]. Later, **cepafunings I**, **II** (**54**), and **III** (**55**) acylpeptides produced by *B. cepacia* CB-3 were described by Shoji et al. [92]. The mixture of cepafungins has moderate inhibitory activity against pathogenic yeast and fungi such as *C. albicans*, *Candida krusei*, *Aspergillus fumigatus*, *Microsporum canis*, and *T. mentagrophytes*. It showed no curative effect in mice infected with *C. albicans*, and there was no activity against bacteria, but it had a moderate effect on prolonging the survival period of mice in which murine lymphatic leukemia P388 cells were implanted [92]. The elucidation of cepafungin structures showed that compound I is identical to **51** (Figure 8) [93]. Later, Schellenberg et al. [94] named the group of cepafungins as glidobactins and, while studying the genes for the synthesis of **51** in S. brevitalea K481-B101, found homologous gene clusters in *B. pseudomallei* and *B. mallei*. The production but not the antimicrobial activity of **53** synthesized by *B. pseudomallei* was reported by Biggins et al. [95]. Moreover, a **53** variant was described as **deoxyglidobactin C**, which contains a lysine instead of a 4-hydroxylisine within the structure. **Occidiofungin A-D** (**56**–**59**) (Figure 8), synthesized by *B. contaminans* MS14, are glycopeptides with antifungal activity inhibiting a large spectrum of fungal pathogens, among them *Alternaria*, *Aspergillus*, *Fusarium*, *Geotrichum*, *Macrophomina*, *Microsporum*, *Penicillum*, *Pythium*, *Rhizoctonia*, *Trichophyton*, and several *Candida* species [96,97,98]. Occidiofungin disrupts fungal membrane morphology and induces apoptosis [96,99]. A recent study identified actin filaments as the primary cellular target of occidiofungin in fungi [98]. It also has antiparasitic activity, damaging the parasite Cryptosporidium parvum [100]. Occidiofungin is also produced by B. *pyrrocinia* Lyc2, having antifungal activity, attacking *Aspergillus*, *Cladosporium*, *Cochilobolus heterostrophus*, *Colletotrichum* acutatum, *Gaeumannomyces graminis*, *Geotrichum candidum*, *Glomerella cingulate* and *Thieloviopsis basicola* [101]. Moreover, occidofungin was tested in toxicological evaluations and it was found to have minimal toxicity in human fibroblasts and has potent anticancer activity [102]. **Cepacidine A_1_** (**60**) **and A_2_** (**61**) (Figure 8) are glycopeptides produced by *B. cepacia* AF 2001. They are highly similar, with molecular weights of 1199 and 1219 Da, respectively [103]. Cepacidine A_2_ contains asparagine, and A_1_ includes b-hydroxy aspargine, which combined have potent antifungal but no antibacterial activity [104]. The cepacidine mixture inhibits *C. albicans*, *C. glabrata*, *Cryptococcus neoformans*, *S. cerevisiae*, *A. niger*, *Microsporum* gypseum, *Epidermophyton floccosum*, *T. mentagrophyte*, *Trichophyton rubrum*, *F. oxysporum*, and *Rhizopus stolonifera*. Moreover, cepacidine A has immunosuppressive action involving in vitro inhibition of the proliferation of *murine lymphocytes* [105]. Cepacidine A has moderate anthelmintic in vitro but not in vivo activity [106]. **AFC-BC11** is a lipopeptide produced by *B. cepacia* BC11 [107]. This compound is involved in the biological control of *R. solani* damping-off in cotton. **Icosalide A1** (**62**) (Figure 8) is an unusual two-tailed lipocyclopeptide antibiotic produced by *B. gladioli* HKI0739, which is active against entomopathogenic bacteria *B. thuringiensis* and *Paenibacillus larvae* and is involved in swarming inhibition [108]. *Burkholderia gladioli* BCC0238 was also found to synthesize (62), but its antimicrobial activity was not tested [109]. This compound was first reported from Aureobasidium and showed activity against *Streptococcus pyogenes* and *E. faecalis* [110]. *Burkholderia thailandensis* produces **bactobolins A-D** (**63**–**66**) (Figure 8), a group of polyketide-peptide molecules, some of which are potent antimicrobials [111]. The production of these compounds is temperature dependent with better results of production at 30 than 37 °C. The purification of the three most abundant bactobolins showed that **63** and **65** have strong activity against bacteria (*Bacillus* cereus, *B. subtilis*, *B. cenocepacia*, *Paraburkholderia kururiensis*, *Burkholderia vietnamiensis*, *Chromobacterium violaceum*, *E. coli*, *Flavobacterium johnsoniae*, *K. pneumoniae*, *Mycobacterium marinum*, *P. aeruginosa*, *Pseudomonas fluorescens*, *Ralstonia pickettii*, *S. Typhimurium*, *S. aureus*, and *Streptococcus pyogenes*) and fibroblasts. **Xylocandins A_1_**, **A_2_**, **B_1_**, **B_2_**, **C_1_**, **C_2_**, **D_1_**, and **D_2_** were isolated from *B. cepacia* ATCC 3927 [112]. Xylocandins are cyclic peptides containing glycine, serine, asparagine, β-hydroxytyrosine, and an unusual amino acid with the formula C_18_H_37_NO_5_. The mixture of each compound showed that xylocandin A_1_ and A_2_ have a potent antifungal activity inhibiting several *Candida* species and dermatophytes such as *T. mentagrophytes*, *T. rubrum*, *Epidermophyton floccosum*, and *M. canis*, but does not inhibit Gram-negative and -positive bacteria, nor vaginitis in a rat model [113]. *Burkholderia cenocepacia* H111 was detected to produce a diazeniumdiolate metalophore compound called **fragin** (**67**) (Figure 8) [114]. When iron was added to the medium, the antifungal activity of strain H111 diminishes, suggesting that the metal chelation is the molecular basis for antifungal activity. Fragin enantiomers inhibit *F. solani*, *B. cereus*, *B. subtilis*, *B. thuringiensis*, *S. aureus*, and *S. cerevisiae*, but no Gram-negative bacteria such as *C. violaceum*, *E. coli*, *Klebsiella oxytoca*, and *P. syringae*. Besides fragin, strain H111 synthesizes a signal molecule called valdiazen, which shares a high degree of structural homology with fragin, but their function is different since valdiazen has no antimicrobial activity. Valdiazen is a diffusible signal that regulates both itself and fragin and the expression of more than 100 genes, representing a novel quorum-sensing signal. **Betulinans** are produced by *B. pseudomallei* K96243 [115]. During the screening of agonists for eukaryotic phosphodiesterase (PDE), the betulinan **BTH-II0204-207:A** (**68**) (Figure 8) compound produced by *B. pseudomallei* K96243 was found. PDE are divided into 11 families. PDE4 has been implicated in inflammation responses across multiple immune cell types; therefore, PDE4 inhibitors have been extensively investigated as potential therapeutic molecules for a number of inflammatory diseases. Bioactivity assays indicated that **68** is a PDE4 inhibitor. Microbial symbionts are often a source of chemicals that can contribute to host defense against antagonists. *Lagria* beetles live in symbiosis with multiple strains of *Burkholderia* that protect their offspring against pathogens. Among them, *B. gladioli* Lv-StB was found to produce the PK **lagriamide** (**69**) (Figure 8), which inhibits *A. niger* and *P. lilacinum* [116]. **Isosulfazecin** (**70**) (Figure 8) is a NRP b-lactam antibiotic produced by *Pseudomonas mesoacidophila* SB-72310 (now *B. ubonensis*) [117,118]. Compound **70** inhibits *S. Typhimurium* and moderately inhibits *E. coli, P. vulgaris, P. mirabilis, S. marcescens, E. faecalis*, and *B. subtilis*. *Burkholderia ubonensis* SB-72310 also produces **bulgecins**, glycopeptides that induce bulge formation in cooperation with b-lactams and enhance the lytic activity of b-lactam-antibiotics, but bulgecins show no antimicrobial activity [119]. *Burkholderia cepacia* CF-66 displays strong antifungal activity against *R. solani*; a compound named **CF66I** was purified and showed inhibition of *F. oxysporum, Fusarium sambucinum, Rosselinia necatrix, Aspergillus flavus, A. niger, Cochilobus carbonum, B. cinerea, Mucor hiemolis, Penicillum chrysogenum, Rhizopus oryzae, C. albicans, C. neoformens, Pichia membranae*, and *S. cerevisiae* but not *E. coli*, *B. subtilis*, or *S. aureus* [120]. *Pseudomonas aeruginosa*, causing nosocomial and wound infections, possess a signal molecule that integrates quorum sensing (QS) and stress response [121]. This integrated QS molecule (IQS) is identical to aeruginaldehyde from *P. fluorescens* [122]. IQS is effectively captured by the NRP siderophore malleobactin produced by *B. thailandensis*, which results in the formation of a rare nitrone bioconjugate called malleonitrone (**71**) (Figure 8) that is active against the IQS producer and therefore has significance from a pharmaceutical perspective [123]. **Spliceostatins** are spliceosome inhibitors, synthesized by a hybrid NRPS-PKS system of the trans-acyl transferase (AT) type, that show promising anticancer activity. *Burkholderia* sp. FERM BP-3421, identified by 16S sequence (KJ364655) as a member of the Bcc, produces the hemiketal spliceostatins, as well as analogs containing a terminal carboxylic acid [124]. Some spliceostatin analogues (**72**–**73**) and their semisynthetic analogues were evaluated in cell proliferation assays against a panel of solid tumor cell lines, showing potent cytotoxicity [125]. **Diketopiperazines** (**DKP**) are NRP-cyclized molecules comprising amino acid bounded by two peptide bonds [126]. *Burkholderia cepacia* CF-66 synthesizes the molecules diketopiperazines cyclo(Pro-Phe), cyclo(Pro-Tyr), cyclo(Ala-Val), cyclo(Pro-Leu), and cyclo(Pro-Val); all of these compounds are both D- and L-type [127]. These DKP showed a negative effect on the candidacidal activity of the culture supernatant extracts. *Burkholderia cepacia* CF-66 lacks the gen cepI that encodes for an acyl homoserine lactone (AHL), which is involved in QS; however, a study with *B. cenocepacia* J2315^T^ showed that new DKP molecules can inhibit CepI in vitro, impairing the ability of *B. cenocepacia* to produce proteases and siderophores and to form biofilms [128].

## 11. Other Antimicrobial Compounds

**Figure 9:**
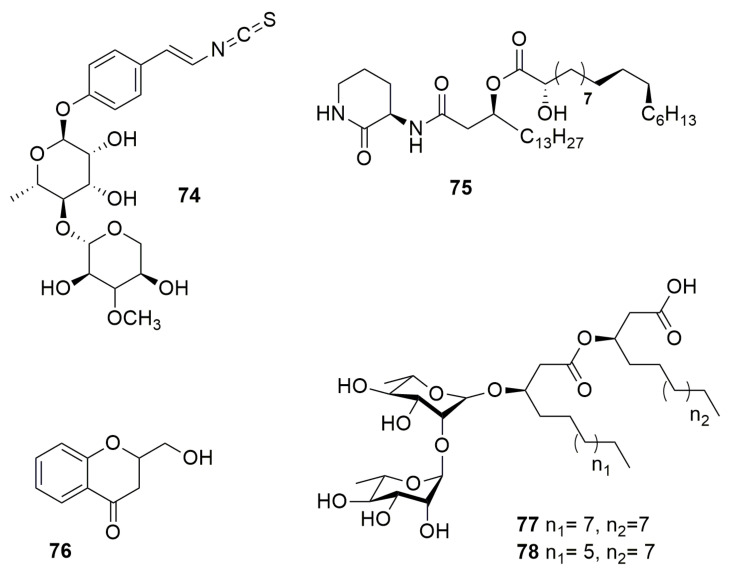
Other antimicrobial compounds. (**74**) Sinapigladioside. (**75**) Cepaciamide A. (**76**) 2-hydroxymethyl-chroman-4-one. (**77**) Di-rhamnolipid C_14_-C_14_. (**78**) Di-rhamnolipids C_12_-C_14_.

**Sinapigladioside** (**74**) (Figure 9), an aromatic glycoside, contains an isothiocyanate moiety, a rare structural feature among bacterial metabolites [51]. This compound produced by *B. gladioli* displays antifungal activity against *P. lilacinum*, which is an egg entomopathogen, *A. fumigatus*, and *Penicillum notatum*. A not fully characterized molecule referred to as “**Compound 1**”, an antimicrobial compound with an unknown structure but with an ion at m/z 391.2845 and produced by *B. orbicola* TAtl-371^T^, was found to inhibit only Tatumella terrea SHS 2008^T^ [129]. **Cepaciamide A** (**75**) (Figure 9) is a (3R, 3′R, 2”R, 5”S, 6”R)-3-N-[3′-(2”-hydroxy-5”, 6”-methylenoctadecanoyl)-hexadecamido]-2-piperidinone isolated from *B. cepacia* D-202 [130]. This compound has toxicity activity against *B. cinerea*, which causes beet root rot in Japan. The bacterial type III secretion system (T3SS) acts as a complex multiunit nanomachine to translocate effector proteins across the bacterial membrane to deliver them directly into eukaryotic host cells [131]. *B. gladioli* NGJ1 produces a prophage tail-like protein (Bg_9562), which is a potential effector secreted by a T3SS and is essential for mycophagy in *R. solani* [132]. Bg_9562 protein showed antifungal activity against *S. cerevisiae*, *C. albicans*, *Alternaria* brassicae, *M. oryzae*, Venturia inaequalis, *F. oxysporum* 7063, *Alternaria* sp., Dedymella sp., Phytophthora sp., *Colletotrichum* sp., Ascochyta rabiei, and Neofusicoccum sp. Moreover, Bg_9562 protein showed no inhibition of *E. coli*, Pantoea ananatis, or B. glaidoli NGJ1. The compound **2-hydroxymethyl-chroman-4-one** (**76**), designated as **MSSP2** (Figure 9), is produced by *Burkholderia* sp. MSSP [133], whose 16S gene sequence (AY551271) indicates that it belongs to the Bcc. The compound MSSP2 displays an inhibitory effect against P. ultimum, *P. capsica*, and S. sclerotiorum. **Altericidins A**, **B**, and **C** produced by *B. cepacia* KB-1 can inhibit a wide range of fungi and yeasts, such as *Alteraria kikuchiana* and *Ustilago maydis*, but has no effect on bacteria [134]. **Rhamnolipids** (Rha) are glycolipidic biosurfactants consisting of rhamnose molecules linked through a β-glycosidic bond to 3-hydroxyfatty acids with various chain lengths produced by bacterial species with several functions such as antimicrobial activity [135]. The non-pathogenic *B. thailandensis* E264^T^ synthesize di-rhamnolipids C_14_-C_14_ (**77**) and C_12_-C_14_ (**78**) (Figure 9), which have antibacterial and antibiofilm activity against *Streptococcus sanguinis*, *Streptococcus oralis*, *Neisseria mucosa*, and *Actinomyces naeslundii* [136]. Other non-pathogenic *Burkholderia* synthesize Rha, such as *B. glumae* and *B. plantarii*; however, their antagonistic activity was not tested [137,138]. Additionally, *B. pseudomallei* produce Rha-Rha C_14_-C_14_, which showed cytotoxic and hemolytic activities [139,140].

## 12. Compounds with Dual Effect

**Figure 10:**
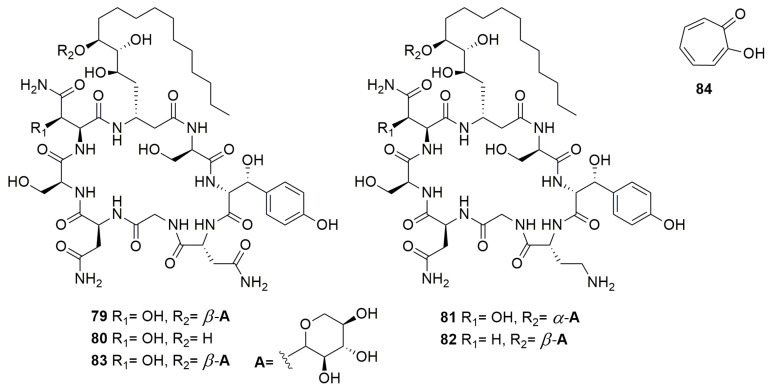
Compounds with dual effect. (**79**–**83**) Burkholdines. (**84**) Tropolone.

Many compounds are beneficial for humans since they are antifungal, antibacterial, or anticancer molecules. However, some of these compounds have a dual effect, both beneficial and toxic. For instance, **burkholdines** (Figure 10) are NRP-cyclic lipopeptides produced by *B. ambifaria* 2.2N with potent antifungal activity [141]. Many burkholdines have been described and the analysis of five representatives (**79**–**83**) showed antifungal activity on *S. cerevisiae*, *C. albicans*, and *A. niger.* However, they also exhibit hemolytic activity [142]. The latter results indicate that these compounds are important for *Burkholderia* virulence. **Tropolone** (**84**) (Figure 10) is a troponoid containing a seven-membered aromatic ring with various substitutions, produced by *B. plantarii* [143]. Tropolone shows broad-spectrum antimicrobial activity against bacteria and fungi, but it is the phytotoxin responsible for rice seedling blight [144,145]. **Cepalycin I** and **cepalycin II** were isolated from *B. cepacia* JN106 [146], but their structure was not reported. These compounds have both hemolytic and antifungal activity, inhibiting *S. cerevisiae*, *C. neoformans*, and *C. albicans*.

## 13. Metabolism as Control

**Figure 11:**
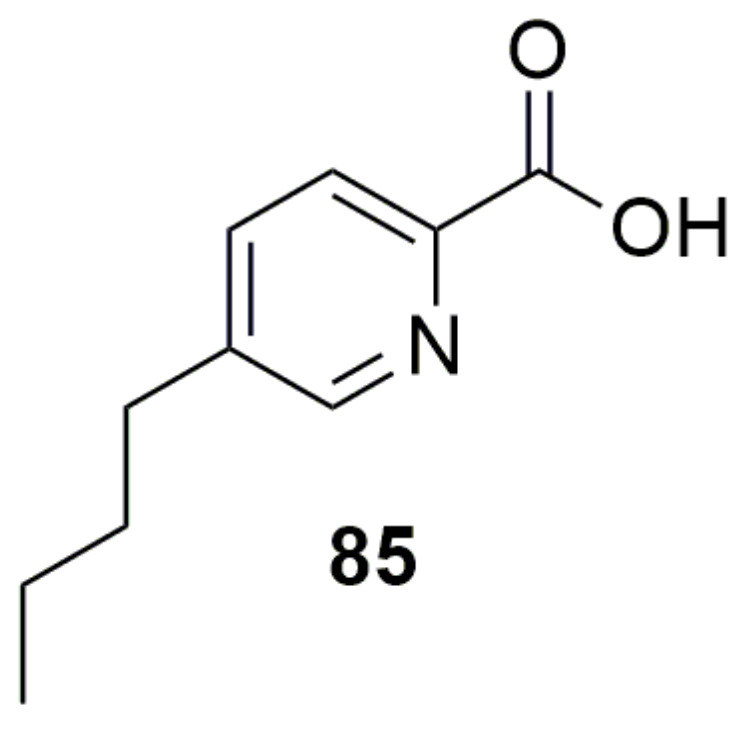
Metabolism as control. (**85**) Fusaric acid.

**Fusaric acid** (**85**) (Figure 11) is a fungal metabolite produced by several *Fusarium* species, which is responsible for wilts and root rot diseases in a number of plants. *Burkholderia ambifaria* T16 can grow with **85** as a sole carbon, nitrogen, and energy source, and showed the ability to detoxify **85** in barley seedlings, suggesting that the strain might serve as a new source of metabolites or genes for the development of novel **85**-detoxification systems [147].

## 14. Data Mining

Genome mining is a promising tool in the search for new bioactive compounds produced by microorganisms. This strategy has been used in several bacterial genomes to analyze their potential as sources of new compounds with pharmacological potential. **Phenazines** are structurally diverse, but all share a conserved seven-gene operon, *phzABCDEFG*, termed the “core phenazine biosynthesis genes” [148]. A genome screening of *Burkholderia* genomes showed that phenazine gene clusters were identified in 20 strains belonging to *B. cepacia*, *Burkholderia lata*, *B. glumae*, B. singularis, *B. ubonensis*, and some *Burkholderia* sp. strains [34]. A genome mining of 64 *B. ambifaria* strains revealed an armory of known and unknown pathways within this species, among them the biosynthetic gene cluster to produce **cepacin**, which was the mode of action for the biopesticidal activity of *B. ambifaria* [48]. In this study [48], other compounds were found in *B. ambifaria* genomes, such as **pyrrolnitrin**, **burkholdines**, **hydroxyquinolines**, **bactobolins**, and **enacyloxina IIa**. Moreover, Mullins and Mahenthiralingam [149] analyzed 4000 genomes representing the genera of *Burkholderia* s.l.; among them, the *Burkholderia* species harbored more biosynthetic gene clusters and the more diverse clusters per species compared to the remaining genera from *Burkholderia* s.l. These clusters include genes involved in the production of **bacteriocins**, **phosphonates**, **lassopeptides**, **NRPS**, **betalactones**, **transAT-PKS**, and **terpenes**. **Chitinases** are glycosyl hydrolases that catalyze the hydrolytic degradation of chitin, one of the major constituents of cell walls of fungi. The genome analysis of *B. orbicola* TAtl-371^T^ showed the presence of the gene BCAL1722 that encodes a chitinase belonging to family 18 of the glycosyl hydrolases, as well as a gene that encodes a predicted chitinase [129]. The authors also found that several *B. cenocepacia* strains contain homologues; however, the activity of chitinase was not present in strain TAtl-371^T^. Moreover, Rojas-Rojas et al. [129] also found that the genome of strain TAtl-371^T^ contains 30 genes reported for the biosynthesis of the bacteriocin **BceTMilo**. A genomic search for **LlpAs** in *Burkholderia* genomes and phylogenetic analysis showed two distinct clusters; one of them belongs to the *B. pseudomallei* group, including *Burkholderia oklahomensis*, *B. pseudomallei*, *B. mallei*, and *B. thailandensis* [73]. Another bacteriocin studied was ColM in *Burkholderia*. The colM-like bacteriocin gene was found mainly in Bcc and *B. oklahomensis* [74]. **Ubonodin** was found in the genome of 16 out of 306 *B. ubonensis* strains, which might be in relation to the intriguingly large size of 28 aa of the core peptides, longer than any previously characterized example [79]. HMAQ produced by the biosynthetic operon named *hmqABCDEFG* was searched for in the genome of Bcc strains [150]. The analysis showed that one-third of Bcc species carry a homolog of the *hmqABCDEFG*, and not all sequenced strains in each species possess this operon.

## 15. Conclusions

The ability of *Burkholderia* to produce antimicrobial compounds is remarkable, not just for the variety of molecules synthesized but also for the diversity of targets they attack, namely bacteria, fungi, cancer cells, tumor cells, or inflammatory processes. The compounds produced belong to a variety of chemical natures, such as N-containing heterocycles, volatile organic compounds, polyenes, polyynes, siderophores, macrolides, bacteriocins, quinolones, non-ribosomal peptides, polyketides, and other unclassified compounds such as sinapigladiosides, cepaciamide A, altericidins, and rhamnolipids, among others. Moreover, there are compounds that have both beneficial and toxic effects, such as burkholdines, tropolone, and others. The mining of genomes is another important method of finding new molecules. Certainly, *Burkholderia* is still a group of bacteria with as-yet unexplored compounds waiting to be discovered. Several papers about new *Burkholderia* strains are published daily, which may contain information about the new antimicrobial compounds they produce that have the potential to be used against multidrug-resistant microorganisms.

## Supplementary Materials

The following supporting information can be downloaded at https://www.mdpi.com/article/10.3390/molecules28041646/s1: Table S1: Antimicrobial compounds produced by *Burkholderia* sensu stricto. References [151,152] are cited in the Supplementary Materials.

## References

[1] Sawana A., Adeolu M., Gupta R.S. (2014). Molecular signatures and phylogenomic analysis of the genus *Burkholderia*: Proposal for division of this genus into the emended genus *Burkholderia* containing pathogenic organisms and a new genus *Paraburkholderia* gen. nov. harboring environmental species. Front. Genet..

[2] Dobritsa A.P., Samadpour M. (2016). Transfer of eleven *Burkholderia* species to the genus *Paraburkholderia* and proposal of *Caballeronia* gen. nov., a new genus to accommodate twelve species of *Burkholderia* and *Paraburkholderia*. Int. J. Syst. Evol. Microbiol..

[3] Lopes-Santos L., Castro D.B.A., Ferreira-Tonin M., Correa D.B.A., Weir B.S., Park D., Mariscal-Ottoboni L.M., Rodrigues-Neto J., Lanza-Destefano S.A. (2017). Reassessment of the taxonomic position of *Burkholderia andropogonis* and description of *Robbsia andropogonis* gen. nov., comb. nov. Anton. Leeuw. Int. J. Gen..

[4] Estrada-de los Santos P., Palmer M., Chavez-Ramírez B., Beukes C., Steenkamp E., Briscoe L., Khan N., Maluk M., Lafos C., Humm E. (2018). Whole genome analyses suggests that *Burkholderia* sensu lato contains two additional novel genera (*Mycetohabitans* gen. nov. and *Trinickia* gen. nov.): Implications for the evolution of diazotrophy and nodulation in the *Burkholderiaceae*. Genes.

[5] Lin Q.H., Lv Y.Y., Gao Z.G., Qiu L.H. (2020). *Pararobbsia silviterrae* gen. nov., sp. nov., isolated from forest soil and reclassification of *Burkholderia alpina* as *Pararobbsia alpina* comb. nov. Int. J. Syst. Evol. Microbiol..

[6] Khakhum N., Tapia D., Torres A.G. (2019). *Burkholderia mallei* and *glanders*. Defense Against Biological Attacks.

[7] Gassiep I., Armostrong M., Norton R. (2020). Human melioidosis. Clin. Microbiol. Rev..

[8] Hall C.M., Baker A.L., Sahl J.W., Mayo M., Scholz H.C., Kaestli M., Schupp J., Martz M., Settles E.W., Busch J.D. (2022). Expanding the *Burkholderia pseudomallei* complex with the addition of two novel species: *Burkholderia mayonis* sp. nov. and *Burkholderia savanae* sp. nov. Appl. Environ. Microbiol..

[9] Pereira-Andrade J., de Souza H.G., Carvalho-Ferreira L., Cnockaert M., De Canck E., Wieme A.D., Peeters C., Gross E., De Souza J.T., Santos-Marbach P.A. (2021). *Burkholderia perseverans* sp. nov., a bacterium isolated from the Restinga ecosystem, a producer of volatile and diffusible compounds that inhibit plant pathogens. Braz. J. Microbiol.

[10] Mahenthiralingam E., Urban T.A., Goldberg J.B. (2005). The multifarious, multireplicon *Burkholderia cepacia* complex. Nat. Rev. Microbiol..

[11] Morales-Ruiz L.M., Rodriguez-Cisneros M., Kerber-Diaz J.C., Rojas-Rojas F.U., Ibarra J.A., Estrada-de los Santos P. (2022). *Burkholderia orbicola* sp. nov., a novel species within the *Burkholderia cepacia* complex. Arch. Microbiol..

[12] Rose H., Baldwin A., Dowson C.G., Mahenthiralingam E. (2009). Biocide susceptibility of the *Burkholderia cepacia* complex. J. Ant. Chemother..

[13] Coenye T., Vandamme P. (2003). Diversity and significance of *Burkholderia* species occupying diverse ecological niches. Environ. Microbiol..

[14] Parke J.L., Gurian-Sherman D. (2001). Diversity of the *Burkholderia cepacia* complex and implications for risk assessment of biological control strains. Ann. Rev. Phytopathol..

[15] Chain P.S.G., Denef V.J., Konstantinidis K.T., Vergez L.M., Agullo L., Reyes V.L., Hauser L., Cordova M., Gomez L., Gonzalez M. (2006). *Burkholderia xenovorans* LB400 harbors a multi-replicon, 9.73-Mbp genome shaped for versatility. Proc. Natl. Acad. Sci. USA.

[16] O’Sullivan L.A., Weightman A.J., Jones T.H., Marchbank A.M., Tiedje J.M., Mahenthiralingam E. (2007). Identifying the genetic basis of ecologically and biotechnologically useful functions of the bacterium *Burkholderia vietnamiensis*. Environ. Microbiol..

[17] Bach E., Pereira-Passaglia L.M., Jiao J., Gross H. (2022). *Burkholderia* in the genomic era: From taxonomy to the discovery of new antimicrobial secondary metabolites. Rev. Microbiol..

[18] Depoorter E., Bull M.J., Peeters C., Coenye T., Vandmme P., Mahenthiralingam E. (2016). *Burkholderia*: An update on taxonomy and biotechnological potential as antibiotic producers. Appl. Microbiol. Biotechnol..

[19] Kerru N., Gummidi L., Maddila S., Gangu K.K., Jonnalagadda S.B. (2020). A review on recent advances in nitrogen-containing molecules and their biological applications. Molecules.

[20] Valenca C.A.S., Barbosa A.A.T., Souto E.B., Caramao E.B., Jain S. (2021). *Volatile nitrogenous* compounds from bacteria: Source of novel bioactive compounds. Chem. Biodivers..

[21] Prasad J., Pandey P., Anand R., Raghuwanshi R. (2021). Drought Exposed *Burkholderia seminalis* JRBHU6 exhibits antimicrobial potential through pyrazine-1,4-dione derivatives targeting multiple bacterial and fungal proteins. Front. Microbiol..

[22] Petri G.L., Spano V., Spatola R., Holl R., Raimondi M.V., Barraja P., Montalbano A. (2020). Bioactive pyrrole-based compound with target selectivity. Eur. J. Med. Chem..

[23] Mitchell R.E., Teh K.L. (2005). Antibacterial iminopyrrolidines from *Burkholderia plantarii*, a bacterial pathogen of rice. Org. Biomol. Chem..

[24] Mitchell R.E., Greenwood D.R., Sarajioni V. (2008). An antibacterial pyrazole derivative from *Burkholderia glumae*, a bacterial pathogen of rice. Phytochemistry.

[25] Pawar S., Chaudhari A., Prabha R., Shukla R., Singh D.P. (2019). Microbial pyrrolnitrin: Natural metabolite with immense practical utility. Biomolecules.

[26] Arima K., Imanaka H., Kousaka M., Fukuta A., Tamura G. (1964). Pyrrolnitrin, a new antibiotic substance produced by *Pseudomonas*. Agric. Biol. Chem..

[27] Kilani J., Fillinger S. (2016). Phenylpyrroles: 30 years, two molecules and (nearly) no resistance. Front. Microbiol..

[28] El-Banna N., Winkelmann G. (1998). Pyrrolnitrin from *Burkholderia cepacia*: Antibiotic activity against fungi and novel activities against streptomycetes. J. Appl. Microbiol..

[29] Sultan M.Z., Park K., Lee S.Y., Park J.K., Varughese T., Moon S.S. (2008). Novel oxidized derivatives of antifungal pyrrolnitrin from the bacterium *Burkholderia cepacia* K87. J. Antibiot..

[30] Jung B.K., Hong S.J., Park G.S., Kim M.C., Shin J.H. (2018). Isolation of *Burkholderia cepacia* JBK9 with plant growth-promoting activity while producing pyrrolnitrin antagonistic to plant fungal diseases. Appl. Biol. Chem..

[31] Webster G., Jones C., Mullins A.J., Mahenthiralingam E. (2020). A rapid screening method for the detection of specialized metabolites from bacteria: Induction and suppression of metabolites from *Burkholderia* species. J. Microbiol. Methods.

[32] Yan J., Liu W., Cai J., Wang Y., Li D., Hua H., Cao H. (2021). Advances in phenazines over the past decade: Review of their pharmacological activities, mechanisms of action, biosynthetic pathways, and synthetic strategies. Mar. Drugs.

[33] Cartwright D.K., Chilton W.S., Benson D.M. (1995). Pyrrolnitrin and phenazine production by *Pseudomonas cepacia*, strain 5.5B, a biocontrol agent of *Rhizoctonia solani*. Appl. Microbiol. Biotechnol..

[34] Hendry S., Steinke S., Wittstein K., Stadler M., Harmrolfs K., Adewunmi Y., Sahukhal G., Elasri M., Thomashow L., Weller D. (2021). Functional analysis of phenazines biosynthesis genes in *Burkholderia* spp. Appl. Environ. Microbiol..

[35] Han J.W., Kim J.D., Lee J.M., Ham J.H., Lee D., Kim B.S. (2014). Structural elucidation and antimicrobial activity of new phencomycin derivatives isolated from *Burkholderia glumae* strain 411gr-6. J. Antibiot..

[36] Xu Z., Wang M., Du J., Huang T., Liu J., Dong T., Chen Y. (2020). Isolation of *Burkholderia* sp. HQB-1, a promising biocontrol bacterium to protect banana against *Fusarium* wilt through phenazine-1-carboxylic acid secretion. Front. Microbiol..

[37] Chen J.H., Xiang W., Cao K.X., Lu X., Yao S.C., Hung D., Huang R.S., Li L.B. (2020). Characterization of volatile organic compounds emitted from endophytic *Burkholderia cenocepacia* ETR-B22 by SPME-GC-MS and their inhibitory activity against various plant fungal pathogens. Molecules.

[38] Xu T., Shi L., Zhang Y., Wang K., Yang Z., Ke S. (2019). Synthesis and biological evaluation of marine alkaloid-oriented β-carboline analogues. Eur. J. Med. Chem..

[39] Huang D., Zhang Z., Li Y., Liu F., Huang W., Min Y., Wang K., Yang J., Cao C., Gong Y. (2022). Carboline derivatives based on natural pityriacitrin as potential antifungal agents. Phytochemi. Lett..

[40] Lin Y.T., Lee C.C., Leu W.M., Wu J.J., Huang Y.C., Meng M. (2021). Fungicidal activity of volatile organic compounds emitted by *Burkholderia gladioli* strain BBB-01. Molecules.

[41] Hunter W.J., Manter D.K. (2014). Antimicrobial properties of an oxidizer produced by *Burkholderia cenocepacia* P525. Curr. Microbiol..

[42] Wu Y., Seyedsayamdost M.R. (2018). The polyene natural product thailandamide A inhibits fatty acid biosynthesis in Gram-positive and Gram-negative bacteria. Biochemistry.

[43] Park J.D., Moon K., Miller C., Rose J., Xu F., Ebmeier C.C., Jacobsen J.R., Mao D., Old W.M., DeShazer D. (2020). Thailandenes, cryptic polyene natural products isolated from *Burkholderia thailandensis* using phenotype-guided transposon mutagenesis. ACS Chem. Biol..

[44] Mahenthiralingam E., Song L., Sass A., White J., Wilmot C., Marchbank A., Boaisha O., Paine J., Knight D., Challis G.L. (2011). Enacyloxins are products of an unusual hybrid modular polyketide synthase encoded by a cryptic *Burkholderia ambifaria* genomic island. Chem. Biol..

[45] Parmeggiani A., Krab I.M., Watanabe T., Nielsen R.C., Dahlberg C., Nyborg J., Nissen P. (2006). Enacyloxin IIa pinpoints a binding pocket of elongation factor Tu for development of novel antibiotics. J. Biol. Chem..

[46] Ross C., Opel V., Scherlach K., Hertweck C. (2014). Biosynthesis of antifungal and antibacterial polyketides by *Burkholderia gladioli* in coculture with *Rhizopus microsporus*. Mycoses.

[47] Parker W.L., Rathnum M.L., Seiner V., Trejo W.H., Principe P.A., Sykes R.B. (1984). Cepacin A and cepacin B, two new antibiotics produced by *Pseudomonas cepacia*. J. Antibiot..

[48] Mullins A.J., Murray J.A.H., Bull M.J., Jenner M., Jones C., Webster G., Green A.E., Neill D.R., Connor T.R., Parkhill J. (2019). Genome mining identifies cepacin as a plant-protective metabolite of the biopesticidal bacterium *Burkholderia ambifaria*. Nat. Microbiol..

[49] Kusumi T., Ohtani I., Nishiyama K., Kakisawa H. (1987). Caryoynencins, potent antibiotics from a plant pathogen. Tetrahedron.

[50] Yamaguchi M., Park H.J., Ishizuka S., Omata K., Hirama M. (1995). Chemistry and antimicrobial activity of caryoynencin analogs. J. Med. Chem..

[51] Florez L.V., Scherlach K., Gaube P., Ross C., Sitte E., Hermes C., Rodrigues A., Hertweck C., Kaltenpoth M. (2017). Antibiotic-producing symbionts dynamically transition between plant pathogenicity and insect-defensive mutualism. Nat. Commun..

[52] Hider R.C., Kong X. (2010). Chemistry and biology of siderophores. Nat. Prod. Rep..

[53] Finking R., Marahiel M.A. (2004). Biosynthesis of nonribosomal peptides. Annu. Rev. Microbiol..

[54] Hur G.H., Vickery C.R., Burkart M.D. (2012). Explorations of catalytic domains in non-ribosomal peptide synthetase enzymology. Nat. Prod. Rep..

[55] Jaremko M.J., Davis T.D., Corpuz J.C., Burkart M.D. (2020). Type II non-ribosomal peptide synthetase proteins: Structure, mechanism, and protein–protein interactions. Nat. Prod. Rep..

[56] Adler C., Corbalan N.S., Seyedsayamdost M.R., Pomares M.F., de Cristobal R.E., Clardy J., Kolter R., Vincent P.A. (2012). Catecholate siderophores protect bacteria from pyochelin toxicity. PLoS ONE.

[57] Ong K.S., Aw Y.K., Lee L.H., Yule C.M., Cheow Y.L., Lee S.M. (2016). *Burkholderia paludis* sp. nov., an antibiotic-siderophore producing novel *Burkholderia cepacia* complex species, isolated from Malaysian tropical peat swamp soil. Front. Microbiol..

[58] Ong K.S., Cheow Y.L., Lee S.M. (2017). The role of reactive oxygen species in the antimicrobial activity of pyochelin. J. Adv. Res..

[59] da Araujo F.D.S., Araujo W.L., Eberlin M.N. (2017). Potential of *Burkholderia seminalis* TC3.4.2R3 as biocontrol agent against *Fusarium oxysporum* evaluated by mass spectrometry imaging. J. Am. Soc. Mass Spectrom..

[60] Meyer J.M., Hohnadel D., Halle F. (1989). Cepabactin from *Pseudomonas cepacia*, a new type of siderophore. J. Gen. Microbiol..

[61] Itoh J., Miyadoh S., Takahasi S., Amano S., Ezaki N., Yamada Y. (1979). Studies on antibiotics BN-227 and BN-227-F, new antibiotics. I. Taxonomy, isolation and characterization. J. Antibiot..

[62] Itoh J., Amano S., Ogawa Y., Kodama Y., Ezaki N., Yamada Y. (1980). Studies on antibiotics BN-227 and BN-227-F, new antibiotics. II. Chemical structure of antibiotics BN-227 and BN-227-F. J. Antibiot..

[63] Darling P., Chan M., Cox A.D., Sokol P. (1998). Siderophore production by cystic fibrosis isolates of *Burkholderia cepacia*. Infec. Immun..

[64] Thomas M.S. (2007). Iron acquisition mechanisms of the *Burkholderia cepacia* complex. BioMetals.

[65] Deng P., Foxfire A., Xu J., Baird S.M., Jia J., Delgado K.H., Shin R., Smith L., Lu S.E. (2017). The siderophore product ornibactin is required for the bactericidal activity of *Burkholderia* contaminans MS14. Appl. Environ. Microbiol..

[66] Rojas-Rojas F.U., Salazar-Gomez A., Vargas-Diaz M.E., Vasquez-Murrieta M.S., Hirsch A.M., De Mot R., Ghequire M.G.K., Ibarra J.A., Estrada-de los Santos P. (2018). Broad-spectrum antimicrobial activity by *Burkholderia cenocepacia* TAtl-371, a strain isolated from the tomato rhizosphere. Microbiology.

[67] Lenz K.D., Klosterman K.E., Mukundan H., Kubicek-Sutherland J.Z. (2021). Macrolides: From toxins to therapeutics. Toxins.

[68] Song L., Jenner M., Masschelein J., Jones C., Bull M.J., Harris S.R., Harkoorn R.C., Vocat A., Romero-Canelon I., Coupland P. (2017). Discovery and biosynthesis of gladiolin: A *Burkholderia gladioli* antibiotic with promising activity against Mycobacterium tuberculosis. J. Am. Chem. Soc..

[69] Riley M.A., Schaechter M. (2009). Bacteriocins, biology, ecology, and evolution. Encyclopedia of Microbiology.

[70] Meade E., Slattery M.A., Garvey M. (2020). Bacteriocins, potent antimicrobial peptides and the fight against multi drug resistant species: Resistance is futile?. Antibiotics.

[71] Yao G.W., Duarte I., Le T.T., Carmody L., LiPuma J.J., Young R., Gonzalez C.F. (2017). A broad-host-range tailocin from *Burkholderia cenocepacia*. Appl. Environ. Microbiol..

[72] Principe A., Fernandez M., Torasso M., Godino A., Fischer S. (2018). Effectiveness of tailocins produced by *Pseudomonas fluorescens* SF4c in controlling the bacterial-spot disease in tomatoes caused by *Xanthomonas vesicatoria*. Microbiol. Res..

[73] Ghequire M.G.K., De Canck E., Wattiau P., Van Winge I., Loris R., Coenye T., De Mot R. (2013). Antibacterial activity of a lectin-like *Burkholderia cenocepacia* protein. MicrobiologyOpen.

[74] Ghequire M.G.K., De Mot R. (2015). Distinct colicin M-like bacteriocin-immunity pairs in *Burkholderia*. Sci. Rep..

[75] Marshall K., Shakya S., Greenhill A.R., Padilla G., Baker A., Warner J.M. (2010). Antibiosis of *Burkholderia ubonensis* against *Burkholderia pseudomallei*, the causative agent for melioidosis. J. Trop. Med. Public Health.

[76] Knappe T.A., Linne U., Zirah S., Rebuffat S., Xie X., Marahiel M.A. (2008). Isolation and structural characterization of capistruin, a lasso peptide predicted from the genome sequence of *Burkholderia thailandensis* E264. Chem. Biol..

[77] Rebufat S., Blond A., Destoumieux-Garzon D., Goulard C., Peduzzi J. (2004). Microcin J25, from the macrocyclic to the lasso structure: Implications for biosynthetic, evolutionary, and biotechnological perspectives. Curr. Protein Pept. Sci..

[78] Knappe T.A., Linne U., Robbel L., Marahiel M.A. (2009). Insights into the biosynthesis and stability of the lasso peptide capistruin. Chem. Biol..

[79] Cheung-Lee W.L., Parry M.E., Zong C., Jaramillo-Cartagena A., Darst S.A., Connell N.D., Rusoo R., Link A.J. (2020). Discovery of ubonodin, an antimicrobial lasso peptide active against members of the *Burkholderia cepacia* complex. ChemBioChem..

[80] Millanao A., Mora A., Villagra N., Bucarey S., Hidalgo A. (2021). Biological effects of quinolones: A family of broad-spectrum antimicrobial agents. Molecules.

[81] Heeb S., Fletcher M.P., Chhabra S.R., Diggle S.P., Williams P., Camara M. (2011). Quinolones: From antibiotics to autoinducers. FEMS Microbiol. Rev..

[82] Wu Y., Seyedsayamdost M.R. (2017). Synergy and target promiscuity drive structural divergence in bacterial alkylquinolone biosynthesis. Cell Chem. Biol..

[83] Wang Y., Hoffmann J.P., Chou C.W., Honer zu Bentrup K., Fuselier J.A., Bitoun J.P., Wimley W.C., Morici L.A. (2020). *Burkholderia thailandensis* outer membrane vesicles exert antimicrobial activity against drug-resistant and competitor microbial species. J. Microbiol..

[84] Yoshihisa H., Sato Z., Hirayama F., Konno K., Shirahama H., Suzui T. (1989). Production of antibiotics by *Pseudomonas cepacia* as an agent for biological control of soilborne plant pathogens. Soil Biol. Biochem..

[85] Saalim M., Villegas-Moreno J., Clark B.R. (2020). Bacterial alkyl-4-quinolones: Discovery, structural diversity and biological properties. Molecules.

[86] Mori T., Yamashita T., Furihata K., Nagai K., Suzuki K.I., Hayakawa Y., Shin-ya K. (2007). Burkholone, a new cytotoxic antibiotic against IGF-I dependent cells from *Burkholderia* sp. J. Antibiot..

[87] Baserga R., Hongo A., Rubini M., Prisco M., Valentinis B. (1997). The IGF-I receptor in cell growth, transformation and apoptosis. Biochim. Biophys. Acta.

[88] Niehs S.P., Kumpfmuller J., Dose B., Little R.F., Ishida K., Florez L.V., Kaltenpoth M., Hertweck C. (2020). Insect-associated bacteria assemble the antifungal butanolide gladiofungin by non-canonical polyketide chain termination. Angew. Chem. Int. Ed. Engl..

[89] Nakou I.T., Jenner M., Dashti Y., Romero-Canelón I., Masschelein J., Mahenthiralingam E., Challis G.L. (2020). Genomics-driven discovery of a novel glutarimide antibiotic from *Burkholderia gladioli* reveals an unusual polyketide synthase chain release mechanism. Angew. Chem. Int. Ed. Engl..

[90] Oka M., Yaginuma K., Numata K., Konishi M., Oki T., Kawaguchi H. (1988). Glidobactins A, B and C, new antitumor antibiotics. II. Structure elucidation. J. Antibiot..

[91] Oka M., Nishiyama Y., Ohta S., Kamei H., Konishi M., Miyaki T., Oki T., Kawaguchi H. (1988). Glidobactins A, B and C, new antitumor antibiotics. I. Production, isolation, chemical properties and biological activity. J. Antibiot..

[92] Shoji J., Hinoo H., Kato T., Hattori T., Hirooka K., Tawara K., Shiratori O., Terui Y. (1990). Isolation of cepafungins I, II and III from *Pseudomonas* species. J. Antibiot..

[93] Terui Y., Nishikawa J., Hinoo H., Kato T., Shoji J. (1990). Structures of cepafungins I, II and III. J. Antibiot..

[94] Schellenberg B., Bigles L., Dudler R. (2007). Identification of genes involved in the biosynthesis of the cytotoxic compound glidobactin from a soil bacterium. Environ. Microbiol..

[95] Biggins J.B., Kang H.S., Ternei M.A., DeShazer D., Brady S.F. (2014). The chemical arsenal of *Burkholderia pseudomallei* is essential for pathogenicity. J. Am. Chem. Soc..

[96] Lu S.E., Novak J., Austin F.W., Gu G., Ellis D., Kirk M., Wilson-Stanford S., Tonelli M., Smith L. (2009). Occidiofungin, a unique antifungal glycopeptide produced by a strain of *Burkholderia contaminans*. Biochemistry.

[97] Ellis D., Gosai J., Emrick C., Heintz R., Romans L., Gordon D., Lu S.E., Austin F., Smith L. (2012). Occidiofungin’s chemical stability and in vitro potency against *Candida* species. Antimicrob. Agents Chemother..

[98] Ravichandran A., Geng M., Hull K.G., Romo D., Lu S.E., Albee A., Nutter C., Gordon D.M., Ghannoum M.A., Lockless S.W. (2018). Occidiofungin, and actin binding antifungal with in vivo efficacy in a vulvovaginal candidiasis infection. bioRxiv.

[99] Emrick D., Ravichandran A., Gosai J., Lu S., Gordon D.M., Smith L. (2013). The antifungal occidiofungin triggers an apoptotic mechanism of cell death in yeast. J. Nat. Prod..

[100] Ma J., Guo F., Jin Z., Geng M., Ju M., Ravichandran A., Orugunty R., Smith L., Zhu G., Zhang H. (2020). Novel antiparasitic activity of the antifungal lead occidiofungin. Antimicrob. Agents Chemother..

[101] Wang X.Q., Liu A.X., Guerrero A., Liu J., Yu X.Q., Deng P., Ma L., Baird S.M., Smith L., Lu S.E. (2016). Occidiofungin is an important component responsible for the antifungal activity of *Burkholderia pyrrocinia* strain Lyc2. J. Appl. Microbiol..

[102] Hing S.L., Ravichandran A., Escano J., Cooley J., Autin F., Lu S.E., Pruett S., Smith L. (2014). Toxicological evaluation of occidiofungin against mice and human cancer cell lines. Sci. Res..

[103] Lim Y., Suh J.W., Kim S., Hyun B., Kim C., Lee C. (1994). Cepacidine A, a novel antifungal antibiotic produced by *Pseudomonas cepacia*. II. Physico-chemical properties and structure elucidation. J. Antibiot..

[104] Lee C.H., Kim S., Hyun B., Suh J.W., Yon C., Kim C., Lim Y., Kim C. (1994). Cepacidine A, a novel antifungal antibiotic produced by *Pseudomonas cepacia*. I. Taxonomy, production, isolation and biological activity. J. Antibiot..

[105] Lee C.H., Suh J.W., Cho Y.H. (1999). Immunosuppressive activity of cepacidine A, a novel antifungal antibiotic produced by *Pseudomonas cepacia*. J. Microbiol. Biotechnol..

[106] Lee C.H., Kempf H.J., Lim Y., Cho Y.H. (2000). Biocontrol activity of *Pseudomonas cepacia* AF2001 and anthelmintic activity of its novel metabolite, cepacidine A. J. Microbiol. Biotechnol..

[107] Kang Y., Carlson R., Tharpe W., Schell M.A. (1998). Characterization of genes involved in biosynthesis of a novel antibiotic from *Burkholderia cepacia* BC11 and their role in biological control of *Rhizoctonia solani*. Appl. Environ. Microbiol..

[108] Dose B., Niehs S.P., Scherlach K., Florez L.V., Kaltenpoth M., Hertweck C. (2018). Unexpected bacterial origin of the antibiotic icosalide: Two-tailed depsipeptide assembly in multifarious *Burkholderia symbionts*. ACS Chem. Biol..

[109] Jenner M., Jian X., Dashti Y., Masschelein J., Hobson C., Roberts D., Jones C., Harris S., Parkhill J., Raja H.A. (2019). An unusual *Burkholderia gladioli* double chain-initiating nonribosomal peptide synthetase assembles “fungal” icosalide antibiotics. Chem. Sci..

[110] Boros C., Smith C.J., Vasina Y., Che Y., Dix A.B., Darveaux B., Pearce C. (2006). Isolation and identification of the icosalides—Cyclic peptolides with selective antibiotic and cytotoxic activities. J. Antibiot..

[111] Chandler J.R., Truong T.T., Silva P.M., Seyedsayamdost M.R., Carr G., Radey M., Jacobs M.A., Sims E.H., Clardy J., Greenberg E.P. (2012). Bactobolin resistance is conferred by mutations in the L2 ribosomal protein. mBio.

[112] Bisacchi G.S., Hockstein D.R., Koster W.H., Parker W.L., Rathnum M.L., Unger S.E. (1987). Xylocandin: A new complex of antifungal peptides. II. Structural studies and chemical modifications. J. Antibiot..

[113] Meyers E., Bissachi G.S., Dean L., Liu W.C., Minassian B., Slusarchyk D.S., Sykes R.B., Tanaka S.K., Trejo W. (1987). Xylocandin: A new complex of antifungal peptides. I. Taxonomy, isolation and biological activity. J. Antibiot..

[114] Jenul C., Sieber S., Daeppen C., Mathew A., Lardi M., Pessi G., Hoepfner D., Neuburger M., Linden A., Gademann K. (2018). Biosynthesis of fragin is controlled by a novel quorum sensing signal. Nat. Commun..

[115] Biggins J.B., Liu X., Feng Z., Brady S.F. (2011). Metabolites from the induced expression of cryptic single operons found in the genome of *Burkholderia pseudomallei*. J. Am. Chem. Soc..

[116] Florez L.V., Scherlach K., Miller I.J., Rodrigues A., Kwan J.C., Hertweck C., Kaltenpoth M. (2018). An antifungal polyketide associated with horizontally acquired genes supports symbiont-mediated defense in *Lagria villosa* beetles. Nat. Commun..

[117] Imada A., Kitano K., Kintaka K., Muroi M., Asai M. (1981). Sulfazecin and isosulfazecin, novel β-lactam antibiotics of bacterial origin. Nature.

[118] Loveridge E.J., Jones C., Bull M.J., Moody S.C., Kahl M.W., Khan Z., Neilson L., Tomeva M., Adams S.E., Wood A.C. (2017). Reclassification of the specialized metabolite producer *Pseudomonas mesoacidophila* ATCC 31433 as a member of the *Burkholderia cepacia* complex. J. Bacteriol..

[119] Imada A., Kintaka K., Nakao M., Shinagawa S. (1982). Bulgecin, a bacterial metabolite which in concert with β-lactam antibiotics causes bulge formation. J. Antibiot..

[120] Quan C.S., Zheng W., Liu Q., Ohta Y., Fan S.D. (2006). Isolation and characterization of a novel *Burkholderia cepacia* with strong antifungal activity against *Rhizoctornia solani*. Appl. Environ. Biotechnol..

[121] Lee J., Wu J., Deng Y., Wang J., Wang C., Wang J., Chang C., Dong Y., Williams P., Zhang L.H. (2013). A cell-cell communication signal integrates quorum sensing and stress response. Nat. Chem. Biol..

[122] Ye L., Cornelis P., Guillemyn K., Ballet S., Christophersen C., Hammerich O. (2014). Structure revision of N-mercapto-4-formylcarbostyril produced by *Pseudomonas fluorescens* G308 to 2-(2-hydroxyphenyl)thiazole-4-carbaldehyde (aeruginaldehyde). Nat. Prod. Commun..

[123] Trottmann F., Franke J., Ishida K., Garcia-Altares M., Hertweck C. (2019). A pair of bacterial siderophores releases and traps an intercellular signal molecule: An unusual case of natural nitrone bioconjugation. Angew. Chem. Int. Ed..

[124] Eustaquio A.S., Janso J.E., Ratnayake A.S., O’Donnell C.J., Koehn F.E. (2014). Spliceostatin hemiketal biosynthesis in *Burkholderia* spp. is catalyzed by an iron/-ketoglutarate-dependent dioxygenase. Proc. Natl. Acad. Sci. USA.

[125] He H., Ratnayake A.S., Janso J.E., Min H., Yang H.Y., Loganzo F., Shor B., O’Donnell J.C., Koehn F.E. (2014). Cytotoxic spliceostatins from *Burkholderia* sp. and their semisynthetic analogues. J. Nat. Prod..

[126] Prasad C. (1995). Bioactive cyclic dipeptides. Peptides.

[127] Wang J.H., Quan C.S., Qi X.H., Li X., Fan S.D. (2010). Determination of diketopiperazines of *Burkholderia cepacia* CF-66 by gas chromatography–mass spectrometry. Anal. Bioanal. Chem..

[128] Scoffone V.C., Chiarelli L.R., Makarov V., Brackman G., Israyilova A., Azzalin A., Forneris F., Riabova O., Savina S., Coenye T. (2016). Discovery of new diketopiperazines inhibiting *Burkholderia cenocepacia* quorum sensing in vitro and in vivo. Sci. Rep..

[129] Rojas-Rojas F.U., Sanchez-Lopez D., Tapia-Garcia E.Y., Arroyo-Herrera I., Maymon M., Humm E., Huntemann M., Clum A., Pillay M., Palaniappan K. (2019). Draft genome of *Burkholderia cenocepacia* TAtl-371, a strain from the *Burkholderia cepacia* complex retains antagonism in different carbon and nitrogen sources. Curr. Microbiol..

[130] Jiao Y., Yoshihara T., Ishikuri S., Uchino H., Ichihara A. (1996). Structural identification of cepaciamide A, a novel fungitoxic compound from *Pseudomonas cepacia* D-202. Tetrahedron.

[131] Deng W., Marshall N.C., Rowland J.L., McCoy J.M., Worrall L.J., Santos A.S., Strynadka N.C.J., Finlay B.B. (2017). Assembly, structure, function and regulation of type III secretion systems. Nat. Rev. Microbiol..

[132] Swain D.M., Yadav S.K., Tyagi I., Kumar R., Kumar R., Ghosh S., Das J., Jha G. (2017). A prophage tail-like protein is deployed by *Burkholderia bacteria* to feed on fungi. Nat. Commun..

[133] Kang J.G., Shin S.Y., Kim M.J., Bajpai V., Maheshwari D.K., Kang S.C. (2004). Isolation and anti-fungal activities of 2-hydroxymethyl-chroman-4-one produced by *Burkholderia* sp. MSSP. J. Antibiot..

[134] Kirinuki T., Ichiba T., Katamaya K. (1984). General survey of action site of altericidins on metabolism of *Alternaria kikuchiana* and *Ustilago maydis*. J. Pestic. Sci..

[135] Abdel-Mawgoud A.M., Lepine F., Deziel E. (2010). Rhamnolipids: Diversity of structures, microbial origins, and roles. Appl. Microbiol. Biotechnol..

[136] Elshikh M., Funston S., Chebbi A., Ahmed S., Marchant R., Banat I.M. (2017). Rhamnolipids from non-pathogenic *Burkholderia thailandensis* E264: Physicochemical characterization, antimicrobial and antibiofilm efficacy against oral hygiene related pathogens. N. Biotechnol..

[137] Hormann B., Muller M.M., Syldatk C., Hausmann R. (2010). Rhamnolipid production by *Burkholderia plantarii* DSM 9509^T^. Eur. J. Lipid Sci. Technol..

[138] Costa S., Deziel E., Lepine F. (2011). Characterization of rhamnolipid production by *Burkholderia glumae*. Lett. Appl. Microbiol..

[139] Funston S.J., Tsaousi K., Rudden M., Smyth T.J., Stevenson P.S., Marchant R., Banat I.M. (2016). Characterizing rhamnolipid production in *Burkholderia thailandensis* E264, a non-pathogenic producer. Appl. Microbiol. Biotechnol..

[140] Haubler S., Nimtz M., Domke T., Wray V., Steinmetz I. (1998). Purification and characterization of a cytotoxic exolipid of *Burkholderia pseudomallei*. Infect. Immun..

[141] Tawfik K.A., Jeffs P., Bray B., Dubay G., Falkinham J.O., Mesbah M., Youssef D., Khalifa S., Schmidt E.W. (2010). Burkholdines 1097 and 1229, potent antifungal peptides from *Burkholderia ambifaria* 2.2N. Org. Lett..

[142] Lin Z., Falkinham J.O., Tawfik K.A., Jeffs P., Bray B., Dubay G., Cox J.E., Schmidt E.W. (2012). Burkholdines from *Burkholderia ambifaria*: Antifungal agents and possible virulence factors. J. Nat. Prod..

[143] Wang M., Tachibana S., Murai Y., Li L., Lau S.Y.L., Cao M., Zhu G., Hashimoto M., Hashidoko Y. (2016). Indole-3-acetic acid produced by *Burkholderia* heleia acts as a phenylacetic acid antagonist to disrupt tropolone biosynthesis in *Burkholderia plantarii*. Sci. Rep..

[144] Azegami K., Nishiyama K., Watanabe Y., Suzuki T., Yoshida M., Nose K., Toda S. (1985). Tropolone as a root growth-inhibitor produced by a plant pathogenic *Pseudomonas* sp. causing seedling blight of rice. Jpn. J. Phytopathol..

[145] Wakimoto S., Hirayae K., Tsuchiya K., Kushima Y., Furuya N., Matsuyama N. (1986). Production of antibiotics by plant pathogenic pseudomonads. Ann. Phytopathol. Soc. Jpn..

[146] Abe M., Nakazawa T. (1994). Characterization of hemolytic and antifungal substance, cepalycin, from *Pseudomonas cepacia*. Microbiol. Immunol..

[147] Simonetti E., Roberts I.N., Montecchia M.S., Gutierrez-Boem F.H., Gomez F.M., Ruiz J.A. (2018). A novel *Burkholderia ambifaria* strain able to degrade the mycotoxin fusaric acid and to inhibit *Fusarium* spp. growth. Microbiol. Res..

[148] Guttenberger N., Blankenfeldt W., Breinbauer R. (2017). Recent developments in the isolation, biological function, biosynthesis, and synthesis of phenazine natural products. Bioorg. Med. Chem..

[149] Mullins A.J., Mahenthiralingam E. (2021). The hidden genomic diversity, specialized metabolic capacity, and revised taxonomy of *Burkholderia* sensu lato. Front. Microbiol..

[150] Coulon P.M.L., Groleau M.C., Deziel E. (2019). Potential of the *Burkholderia cepacia* complex to produce 4-hydroxy-3-methyl-2-alkyquinolines. Front. Cell. Infect. Microbiol..

[151] Matthew A., Jenul C., Carlier A.L., Eberl L. (2016). The role of siderophores in metal homeostasis of member of the genus *Burkholderia*. Environ. Microbiol. Rep..

[152] Li X., Quan C.S., Fan S.D. (2007). Antifungal activity of a novel compound from *Burkholderia cepacia* against plant pathogenic fungi. Lett. Appl. Microbiol..

