# Supplementary material for: Compilation of the Antimicrobial Compounds Produced by Burkholderia Sensu Stricto
Source: Molecules. 2023 Feb 8;28(4):1646. doi: 10.3390/molecules28041646 (PMC9958762; doi:10.3390/molecules28041646)
Supplement: Supplementary file 1 [file molecules-28-01646-s001.zip › molecules-2176071.pdf]

Table S1. Antimicrobial compounds produced by Burkholderia sensu stricto.

Compound (s) number(s)	Compounds	Producing species	Target organisms	Other asignation	Activity value (MIC, IC50, volume, amount, hemolytic units, ug/mL per agar well or per disc, etc.)	Mode of action	Reference
			**N-CONTAINING HETEROCYCLES**				
**Pyrazine-derived**							
1	PPDH					Cell wall/membrane/envelope biogenesis, signal transduction mechanisms, inorganic ion transport and metabolism, secondary metabolites biosynthesis, transport and catabolism, post-translational modification, protein turnover and amino acid transport and metabolism	
**2**	PPDHMP	*Burkholderia seminalis* JRBHU6	*Fusarium oxysporum, Aspergillus niger, Microsporum gypseum, Trichophyton mentaprophytes, Trichoderma harzianum and the bacteria Staphylocccus aureus, Pseudomonas aeruginosa, Escherichia coli, Shigella boydii, Klebsiella pneumoniae*	VOC	MIC: 50 µg/ml of crude methanolic extract		[21]
**Iminomyrrolidines**							
3	v-pyrrolidine	*Burkholderia plantarii* 9424	*Erwinia amylovora*		1 µg		[23]
4	t-pyrrolidine				1 µg		
5	Pyrazole	*Burkholderia glumae* 3729, 8657	*Erwinia amylovora, Pectobacterium carotovorum, Pectobacterium carotovorum* pv atroseptica, *Pectobacterium carotovorum* pv carotovorum, *Pseudomonas syringae* pv syringae, *Xanthomonas campestris* pv pruni		1 µg		[24]
6	Pyrrolnitrin	*Burkholderia pyrrocinia, Burkholderia cepacia, Burkholderia ambifaria*	*Penicillium, Phytophthora capsici, Fusarium oxysporum, Rhizoctonia solani, Colletotrichum gloeosporioides, Sclerotinia sclerotorium, Candida albicans, Hansenula anomala, Saccharomyces cerevisiae, Bacillus, Streptomyces*	Halometabolite	MIC: 2-200 $\mu g.mL^{-1}$ / 30-250 µg/disc	Inhibitory effect on the electron transport system.	[28,29,30,31]
7	Phenazine	*Burkholderia cepacia* 5.5B	*Rhizoctonia solani*	Involved in biofilm formation by Bcc	10 mg		[33,34]
8	Phencomycin	*Burkholderia glumae* 411gr-6	*Botritys cinerea, Colletotrichum orbiculare, Diaporthe citri, Magnoporte oryzae, Saccharomyces cerevisiae, Bacillus megaterium, Micrococcus luteus, Xanthomonas campestris*	Phenazin with two substituents	MIC: 32-128 $\mu g.mL^{-1}$		[35]
9	Hydroxyphencomycin		*Bacillus megaterium*	Phencomycin derivative	MIC: 128 $\mu g.mL^{-1}$		
10	Dihydroxyphencomycin		*Alternaria brassicicola, Aspergillus oryzae, Botrytis cinerea, Cladosporium cucumerinum, Colletotrichum gloeosporioides, Colletotrichum orbiculare, Cylindrocarpon destructans, Diaporthe citri, Fusarium oxysporum, Magnaporthe oryzae, Phytophthora capsici, Rhizopus stolonifer, Sclerotinia sclerotiorum, Candida albicans, Saccharomyces cerevisiae, Escherichia coli, Pseudomonas syringae, Ralstonia solanacearum, Xanthomonas campestris*	Phencomycin derivate	MIC: 1-128 $\mu g.mL^{-1}$		
11	PCA	*Burkholderia* sp. HQB-1	*Fusarium oxysporum, Colletotrichum gloeosporioides, Botrytis cinerea, Curvularia fallax*	Phenazine derivate	MIC: 1.56-6.12 $\mu g.mL^{-1}$		[36]
12	Indole	*Burkholderia cenocepacia* ETR-B22	*Alternaria alternata, Aspergillus niger, Bipolaris sorokiniana, Botrytis cinerea, Fusarium solani, F. oxysporum, Fusarium fujikuroi, Helminthosporium torulosum, Mycosphaerella fijensis, Magnaporthe oryzae, Phyllosticta zingiberi, R. solani*	VOC	50 µL/petri dish	Inhibition of mycelial growth	[37]
13	Pityriacitrin	*Burkholderia* sp. NBF227	Cytotoxic activity against cancer lines	β-carboline alkaloid	IC_{50}: 75.79 µM	Cytotoxic activity against cancer lines	[38]

14	Pityriacitrin B	*Burkholderia* sp. NBF227	Cytotoxic activity against cancer lines	β-carboline alkaloid	IC_{50}: 41.88-67.69 µM	Cytotoxic activity against cancer lines	[38]
			**VOLATILE ORGANIC COMPOUNDS**				
15<br>16<br>17<br>18<br>19<br>20	Methyl anthranilate<br>Methyl salicylate<br>Methyl benzoate<br>Benzyl propionate<br>Benzyl acetate<br>3,5-Di-*tert*-butylphenol		*Alternaria alternata, Aspergillus niger, Bipolaris sorokiniana, Botrytis cinerea, Fusarium solani, Fusarium oxysporum, Fusarium fujikuroi, Helminthosporium torulosum, Mycosphaerella fijensis, Magnoporte oryzae, Phyllosticta zingiberi, Ralstonia solani*				
21	Allyl benzyl ether		*Alternaria alternata, Aspergillus niger, Bipolaris sorokiniana, Botrytis cinerea, Fusarium fujikuroi, Helminthosporium torulosum, Mycosphaerella fijensis, Magnoporte oryzae, Phyllosticta zingiberi, Rhizoctonia solani*				
22	Benzyl benzoate	*Burkholderia cenocepacia* ETR-B22	*Aspergillus niger, Rhizoctonia solani*		50 µL/petri dish	Inhibition of mycelial growth	[37]
23	Dimethyl trisulfide		*Alternaria alternata, Aspergillus niger, Bipolaris sorokiniana, Botrytis cinerea, Fusarium solani, Fusarium oxysporum, Fusarium fujikuroi, Helminthosporium torulosum, Mycosphaerella fijensis, Magnoporte oryzae, Phyllosticta zingiberi, Ralstonia solani*				
24	Nonaoic acid		*Alternaria alternata, Aspergillus niger, Bipolaris sorokiniana, Botrytis cinerea, Fusarium solani, Fusarium oxysporum, Fusarium fujikuroi, Helminthosporium torulosum, Mycosphaerella fijensis, Magnoporte oryzae, Phyllosticta zingiberi, Ralstonia*				
25	2-pentadecanone		*Bipolaris sorokiniana, Rhizoctonia solani*				
26	3-hexen-1-ol, benzoate, (*Z*)-		*Alternaria alternata, Botrytis cinerea, Magnoporte oryzae*				
27<br>28	Dimethy disulfide<br>2,5-dimethylfuran	*Burkholderia gladioli* BBB-01	*Magnaporthe oryzae, Gibberella fujikuroi, Sarocladium oryzae, Phellinus noxius* and *Colletotrichum fructicola, Candida albicans*		1-100 µL		[40]
			**POLYENES**				
	Polyene	*Burkholderia cenocepacia* P525	*Enterobacter soli, Enterobacter aerogoenes*	Oxidizer	50 µL	Bacteriostatic effect by extending the lag phase of growth	[41]
29	Thailandamide A	*Burkholderia thailandensis* DW503	*Bacillus subtilis, Staphylococcus aureus, Neisseria gonorrhoeae, Escherichia coli, Vibrio parahaemolyticus, Klebsiella pneumoniae, Acinetobacter baumannii, Saccharomyces cerevisiae*		MIC: 1-50 µM	Inhibition of the first step of fatty acid biosynthesis by targeting acetyl-CoA carboxylase (AccA)	[42]
30	Thailandenes A		*Staphylococcus aureus, Bacillus subtilis, Saccharomyces cerevisiae*		IC_{50}: 7.9-10.8 µM	Induces oxidative stress in sensitive cells, and formation of reactive oxygen species*	[43]
31	Thailandenes B		*Staphylococcus aureus, Saccharomyces cerevisiae*		IC_{50} 4-9 µM		
32	Thailandenes C			PK			
33, 34	Enacyloxin lla, iso-enacyloxina lla	*Burkholderia ambifaria* AMMD^{T}, *Burkholderia gladioli* pv. *cocovenens* ATCC 33664^{T}	*Burkholderia multivorans, Burkholderia dolosa, Acinetobacter baumannii*		MIC: 3-15 $mg.L^{-1}$	Inhibit protein biosynthesis by binding to ribosomal elongation factor Tu	[44]
35	Enacyloxin IIIa	*Burkholderia gladioli* pv. *cocovenens* ATCC 33664^{T}	*Escherichia coli, Pseudomonas aeruginosa*		50 $\mu g.mL^{-1}$		[46]
			**POLYYNES**				
36	Cepacin A	*Burkholderia cepacia* SC 11,783, *Burkholderia ambifaria* J82/BCC0191, *Burkholderia ambifaria* M54/BCC0316	*Staphylococcus aureus, Micrococcus luteus, Escherichia coli, Proteus vulgaris, Salmonella typhosa, Pythium ultimum*	Acetylenic antibiotic	MIC: 0.1-25 $\mu g.mL^{-1}$	Inhibition of protein synthesis by binding to the 50S ribosomal subunit	[47,48]
37	Cepacin B	*Burkholderia cepacia* SC 11,783	*Staphylococcus aureus, Microbacterium luteus, Escherichia coli, Klebsiella aerogenes, Proteus mirabilis, Proteus rettgeri, Proteus vulgaris, Salmonella typhi, Shigella sonnei, Enterobacter cloacae, Enterobacter aerogenes, Citrobacter freundii*		MIC: 0.05-25 $\mu g.mL^{-1}$		[47]
38	Caryoynecin A	*Burkholderia*	*Staphylococcus aureus, Bacillus subtilis, Enterococcus faecalis, Escherichi coli,*	Fatty acid-derived	MIC: 0.02-25 $\mu g.mL^{-1}$		[50,51]

		caryophylli, Burkholderia gladioli Lv-StA	*Salmonella enteritidis, Klebsiella penaumoniae, Serratia marcescens, Proteus vulgalis, Shigella flexneri, Enterobacter cloacae, Pseudomonas aeruginosa, Tricophyton mentagrophytes, Tricophyton interdigitale, Tricophyton rubrum, Purpureocillium lilacinum*				
39	Caryoynecin B	*Burkholderia caryophylli*	*Staphylococcus aureus*, *Bacillus subtilis, Enterococcus faecalis, Escherichi coli, Salmonella enteritidis, Klebsiella penaumoniae, Serratia marcescens, Proteus vulgalis, Shigella flexneri, Enterobacter cloacae, Pseudomonas aeruginosa, Tricophyton mentagrophytes, Tricophyton interdigitale, Tricophyton rubrum*				
40	Caryoynecin C	*Burkholderia caryophylli*			MIC: 0.02-0.63 µg.mL^{-1}		
			**SIDEROPHORES**				
41	Pyochelin	"*Burkholderia paludis*", *Burkholderia seminalis* TC3.4.2R3	*Enterococcus faecalis, Staphylococcus aureus, Fusarium oxysporum*	NRP	MIC: 3.13-6.26 µg.mL^{-1}	Production of intracelluar reactive oxygen species leading to disrupt bacterial membrane	[57,59]
42	Cepabactin	*Burkholderia cepacia* ATCC 25416^{T}	*Staphylococcus aureus, Staphylococcus epidermidis, Streptococcus faecalis, Bacillus subtilis, Bacillus anthracis, Escherichia coli, Salomonella* Typhi, *Salmonella* Typhimurium, *Klebsiella pneoumoniae, Proteous vulgaris, Proteus mirabilis, Proteus rettgeri*		MIC: 1.56-50 µg.mL^{-1}		[60,61,62]
43	Ornibactin	*Burkholderia contaminans* MS14	*Xanthomonas citri* pv. malvacearum, *Pectobacterium carotovorum* supsp. carotovorum, *Ralstonia solanacearum, Pseudomonas syringae* pv. syringae, *Erwinia amylovora, Escherichia coli, Clavibacter michiganensis* subsp. michiganensis, *Bacillus megaterium*	NRP	ND	Promotes iron uptake in iron-deficient cells/ protect the cells from metal toxicity and thus play an alternative role in metal homeostasis	[65,151]
			**MACROLIDES**				
44	Gladiolin	*Burkholderia gladioli* BCC0238	*Mycobacterium tuberculosis, Klebsiella pneumoniae, Acinetobacter baumannii, Pseudomonas aeruginosa, Enterobacter clocae, Serratia plymuthica, Ralstonia mannitolilytica*, *Burkholderia multivorans, Escherichia coli, Enterococcus faecium, Staphylococcus aureus, Bacillus subtilis, Candida albicans*	PK	MIC: 4-64 µg.mL^{-1}	Inhibition of RNA polymerase	[68]
45	Lagriene	*Burkholderia gladioli* Lv-StA	*Bacillus thuringiensis, Mycobacterium vaccae, Enterococcus faecalis, Staphylococcus aureus*	PK	50 µg	Inhibition of RNA polymerase	[51]
**BACTERIOCINS**							
	Tailocin (BceTMilo)	*Burkholderia cenocepacia* BC0425	Bcc strains, *Burkholderia gladioli, Burkholderia glumae*		Killing units: 3.86-12.30	Inject ions through the cell membrane and disrtupt the the proton motive force	[72,73]
	Lectin-like (LlpA)	*Burkholderia orbicola* TAtl-371^{T}, AU1054	*Burkholderia ambifaria, Burkholderia anthina, Burkholderia cenocepacia, Burkholderia contaminans, Burkholderia metallica*		10 µg	Interferes with the integration and folding of outer membrane proteins by recognition of BamA receptors	[66,73]
	Burkhocins M1 and M2	*Burkholderia ambifaria* MEX-5, AMMD^{T}	Bcc strains	RiPP	20 µM	Degradation of cell wall precursor lipid II in target cells	[74]
	Bacteriocin-like inhibitory substance (BLIS)	*Burkholderia ubonensis* A21	*Burkholderia pseudomallei*		ND		[75]
	Capistruin (lasso peptide)	*Burkholderia thailandensis* E264	*Paraburkholderia caledonica, Escherichia coli, Pseudomonas aeruginosa*		5 nM	Inhibition of RNA polymerase	[76,78]
	Ubonodin (lasso peptide)	*Burkholderia ubonensis* MSMB2207	*Burkholderia cepacia, Burkholderia multivorans, Escherichia coli*		MIC: 3.9-40 µM	Inhibition of RNA polymerase	[79]
			**QUINOLONES**				
46	HMNQ			Alkylquinolone	IC$_{50}$: 0.3-1.1 µM	Inhibition of pyrimidine biosynthesis	[82]
47	HQNO	*Burkholderia thailandensis* E264	*Bacillus subtilis, Escherichia coli*	Alkylquinolone	IC$_{50}$: 4.1-9.1 µM	Inhibits the cytochrome bc_1 complex of the electron transport chain of oxidative phosphorylation	[82]

48	C7Δ2	*Burkholderia cepacia* RB425, *Burkholderia cepacia PC-II*	*Verticillium dahlia*, *Pyricularia oryzae, Cochliobolus myyabeanus, Rhizoctonia solani, Fusarium oxysporum, Gaeumannomyces graminis, Corynebacterium michiganense* and oomycetes *P. capsici, Pythium ultimum, Fusarium oxysporum, Rhizoctonia solani*	Alkylquinolone	0.1-10 µg.mL^{-1}	Inhibition of pyrimidine biosynthesis	[84,85]
49	Burkholone	*Burkholderia* sp. QN15488	Induces cell death in 32D/GR15 cells in IGF-I containing medium	Quinolone derivative	IC_{50}: 160 nM		[86]
**OTHER NPR-PK COMPOUNDS**							
50	Gladiostatin	*Burkholderia gladioli* BCC0238, BCC1622	*Saccharomyces cerevisiae;* cancer cell lines such as ovarian, pancreatic and colon cancer, inhibits tumor cell migration	PK	*S. cerevisiae* MIC: 4 µg.mL^{-1}; cancer cell lines IC_{50}: 0.24-1.4 µM	Inhibit eukaryotic translation by blocking the binding of tRNA to the E-site of the 60S ribosomal subunit and inhibit tumour cell-migration	[89]
50	Gladiofungin A	*Burkholderia gladioli* HKI0739	*Penicillium notatum*, *Sprobolomyces salmonicolor, Purpureocillium lilacinum*	PK	1-1000 µg.mL^{-1}	Inhibit eukaryotic translation by blocking the binding of tRNA to the E-site of the 60S ribosomal subunit and inhibit tumour cell-migration*	[88]
51	Glidobactin A	*Burkholderia pseudomallei, Burkholderia mallei*			MIC: 1.6-50 µg.mL^{-1}		[90]
52	Glidobactin B	*Schlegelella brevitalea* K481-B101 (member of Burkholderiales)	*Candida albicans, Cryptococcus neoformans, Aspergillus fumigatus, Aspergillus flavus, Trichophyton mentagrophytes, Blastomyces dermatitides, Mucor spinosus*		MIC: 0.8-50 µgmL^{-1}	Inhibition of protein biosynthesis	[90]
53	Glidobactin C	*Burkholderia pseudomallei*		NRP-PK	MIC: 0.2-50 µg.mL^{-1}		[90,95]
54	Cepafungin I	*Burkholderia cepacia* CB-3					[92]
54	Cepafungin II	*Burkholderia cepacia* CB-3, *Burkholderia pseudomallei, Burkholderia mallei*	*Candida albicans, Candida krusei, Aspergillus fumigatus, Microsporum canis, Trichophyton mentagrophytes*		MIC: 1.6-12.5 µg.mL^{-1}	Prolongs the survival period of mice implanted with P388 marine lymphoid leukemia cells. However, the mechanism of action as an antifungal agents remains to be determined	[92,93,94]
55	Cepafungin III	*Burkholderia cepacia* CB-3					[92]
56, 57, 58, 59	Occidiofungin	*Burkholderia contaminans* MS14	*Alternaria, Aspergillus, Fusarium, Geotrichum, Macrophomina, Microsporum, Penicillum, Pythium, Rhizoctonia, Trichopyton,* several *Candida* species, *Cryptosporidium parvum*	NRP-PK	MIC: 0.5-32 µg.mL$^{-1/}$ *Cryptosporidium parvum in vitro* with limited cytotoxicity (50% effective concentration [EC_{50}]: 120 nM versus 50% cytotoxic concentration [TC_{50}]= 988 nM)	Disrupts fungal membrane morphology and induces apoptosis	[96,97,99]
		Burkholderia pyrrocinia Lyc2	*Aspergillus, Cladosporium, Cochilobolus heterostrophus, Colletotrichum acutatum, Gaeumannomyces graminis, Geotrichum candidum, Glomerella cingulate, Thieloviopsis basicola*		ND		[101]
60, 61	Cepacidine A_1 and A_2	*Burkholderia cepacia* AF 2001	*Candida albicans, Candida glabrata, Cryptococcus neoformans, Saccharomyces cerevisiae, Aspergillus niger, Microsporum gypseum, Epidermophyton floccosum, Trichophyton mentagrophyte, Trichophyton rubrum, Fusarium oxysporum, Rhizopus stolonifera*	NRP-PK	MIC: 0.013-0.391 µg.mL^{-1}	Disrupts fungal membrane morphology and induces apoptosis	[103,104]

	AFC-BC11	*Burkholderia cepacia* BC11	*Rhizoctonia solani, Pythium ultimum, Colletotrichum, Helminthosporium maydis, Botrytis cinerea, Fusarium, Rhizopus stolonifer, Rhodotorula glutinis, Sclerotium rolfsii, Scopulariopsis brevicaulis*	NRP-PK	MIC: 0.4-10.8 μg.mL^{-1}	Binding to the phosphate groups of LPS of the outer and inner membrane	[107]
62	Icosalide A1	*Burkholderia gladioli* HKI0739	*Bacillus thuringiensis, Paenibacillus larvae*	NRP	MIC: 3.1-12.5 μg.mL^{-1}	Involved in inhibiting swarming	[108]
		Burkholderia gladioli BCC0238	*Candida albicans, Enterococcus faecium, Streptococcus pyogenes*	NRP	MIC: 8-16 μg.mL^{-1}	Binding to the phosphate groups of LPS of the outer and inner membrane	[109]
63	Bactobolin A				MIC: 0.19-50 μg.mL-1		
65	Bactobolin C	*Burkholderia thailandensis*	*Bacillus cereus, Bacillus subtilis, Burkholderia cenocepacia, Paraburkholderia kururiensis, Burkholderia vietnamiensis, Chromobacteriu violaceum, Escherichia coli, Flavobacterium johnsoniae, Klebsiella pneumoniae, Mycobacterium marinum, Pseudomonas aeruginosa, Pseudomonas fluorescens, Ralstonia pickettii, Salmonella enteria serovar Typhimurium, Staphylococcus aureus, Streptococcus pyogenes*	PK	MIC: 1.56-50 μg.mL^{-1}	Inhibition of peptide chain elongation by binding to peptidyl transferase on the ribosome	[111]
	Xylocandins A1 and A2	*Burkholderia cepacia* ATCC 3927	*Candida* species, *Trychophyton mentagrophytes*, *Trycophyton rubrum*, *Epidermophyton floccosum, Microsporum canis*	NRP-PK	MIC: 0.05-0.4 μg.mL^{-1}	Binding to the phosphate groups of LPS of the outer and inner membrane	[113]
67	Fragin	*Burkholderia cenocepacia* H111	*Fusarium solani, Bacillus cereus, Bacillus subtilis, Bacillus thuringiensis, Staphylococcus aureus, Saccharomyces cerevisiae*	NRP	20-80 μg		[114]
68	BTH-II0204-207:A	*Burkholderia pseudomallei* K96243, *Burkholderia thailandensis*	*Bacillus subtilis, Saccharomyces cerevisiae*	NRP	MIC: 11-33 μg.mL^{-1}	Inhibition of type-4 phosphodiesterases (PDE4)	[115]
69	Lagriamide	*Burkholderia gladioli* Lv-StB	*Aspergillus niger, Purpureocillium lilacinum*	PK	50 μg	Similar to bistramides	[116]
70	Isosulfazecin	*Burkholderia ubonensis*	*Salmonella enterica* serovar Typhimurium, *Escherichia coli*, *Proteus vulgaris*, *Proteus mirabilis*, *Serratia marcescens*, Enterococcus *faecalis, Bacillus subtilis*	NRP	MIC: 0.78-100 μg.mL^{-1}	Inhibition of cell wall biosynthesis	[117,118]
72,73	Spliceostatins	*Burkholderia thailandensis*, *Burkholderia* sp. FERM BP-3421	Potent cytotoxicity against tumor cell lines	NRP-PK	IC$_{50}$: 0.11-950 nM	Spliceosome inhibitor	[124,125]
	Diketopiperazines	*Burkholderia cepacia* CF-66, *Burkholderia cenocepacia* J2315^{T}	*Candida albicans*	NRP	1 mL	Cyclic dipeptides	[126,127]
			**OTHER ANTIMICROBIAL COMPOUNDS**				
74	Sinapigladioside	*Burkholderia gladioli* Lv-StA	*Purpureocillum lilacinum*, *Aspergillus fumigatus, Penicillum notatum*	Isothiocyanate moiety	50 μg		[51]
	Compound 1	*Burkholderia orbicola* TAtl-371^{T}	*Tatumella terrea* SHS 2008^{T}		10 μg		[66]
75	Cepaciamide A	*Burkholderia cepacia* D-202	*Botritys cinerea*		100 ppm		[130]
	Bg_9562 protein	*Burkholderia gladioli* NGJ1	*Saccharomyces cerevisiae, Candida albicans*, *Alternaria brassicae, Magnoporte oryzae, Venturia inaequalis, Fusarium oxysporum*, *Alternaria* sp., *Dedymella* sp., *Phytophthora* sp., *Colletotrichum* sp.,	Prophage tail-like	MIC: 15 μg.ml^{-1}	Mycophagy, causes hyphal disintegration	[132]
76	MSSP2	*Burkholderia* sp.	*Ascochyta rabiei, Neofusicoccum* sp. *Pithyum ultimum, Phytophthora capsici* and *Sclerotinia sclerotiorum*		ED$_{50}$: 35.7-54.9 ppm		[133]
	Altericidins A, B and C	*Burkholderia cepacia* KB-1	*Alteraria kikuchiana, Ustilago maydis*	Peptide antibiotics	8-50 ppm	Inhibits the transport of precursors for the biosynthesis of the fungal cytoplasmic membrane	[134]
	Bulgecins	*Burkholderia*	No antimicrobial activity/synergism with B-lactam anntibiotics	Glycopeptide	1-1000 μg.ml^{-1}	Induces bulge formation acting in	[119]

		ubonensis SB-72310				synergism with β-lactamic antibiotics without binding to PBPs	
	CF66I	*Burkholderia cepacia* CF-66	*Rhizoctonia solani, Fusarium graminearum, Fusarium moniliforme, Fusarium oxysporum, Fusarium sambucinum, Fusarium semitectum, Fusarium solani, Rosselinia necatrix, Aspergillus flavus, Aspergillus niger, Cochilobus carbonum, Botrytis cinerea, Mucor hiemolis, Penicillum chrysogenum, Rhizopus oryzae, Candida albicans, Crytococcus meoformens, Pichia membranae, Saccharomyces cerevisiae, Alternaria alternata, Bipolaris sorokiniana, Colletotrichum lindemuthianum, Curvularia lunata, Monochaetia hirta*		MIC: 2.5-29 $\mu g.ml^{-1}$	Reduced the extension rates of hyphae and induced changes in their morphology, forming multiple branches	[120,152]
71	Malleonitrone	*Burkholderia thailandensis* E264	*Pseudomonas aeruginosa, Bacillus subtilis, Staphyloccous aureus, Enteroccous faecalis, Candida albicans*	Nitrone conjugated	MIC: 10-22 $\mu g.ml^{-1}$	Active against the integrated QS molecule (IQS)	[123]
			**RHAMNOLIPIDS**				
77	di-rhamnolipids C_{14}-C_{14}	*Burkholderia thailandensis* E264, *Burkholderia kururiensis* KP23, *B. glumae* AU6208, *Burkholderia pseudomallei,*	*Streptococcus sanguinis, Streptococcus oralis, Neisseria mucosa, Actinomyces naeslundii/* Citotoxyc effect on fhagocytic (HL60) and nonphagocytic (HeLa) cell lines		MIC: 0.15-1.25 $mg.ml^{-1}$/Citotoxic effects: 20-80 hemolytic units	Surface activity, wetting ability, detergency/ promote the uptake and biodegradation of poorly soluble substrates, act as immune modulators and virulence factors and are involved in surface motility and in bacterial biofilm development/ Induction of reactive oxygen species/ Tensoactive properties/ Cytotoxic and hemolytic activity	[136,137,139,140]
78	di-rhamnolipids C_{12}-C_{14}	*Burkholderia thailandensis E264 Burkholderia plantarii* DSM 9509^T, *Burkholderia glumae* AU6208	ND		ND		[137,138]
			**COMPOUNDS WITH DUAL EFFECT**				
79, 80, 81, 82, 83	Burkholdines	*Burkholderia ambifaria* 2.2N	*Saccharomyces cerevisiae, Candida albicans*, *Aspergillus niger,* hemolytic activity	NRP-PK	MIC: 0.1-31$\mu g.mL^{-1}$/Hemolytic activity: 4.5-37 $\mu g.mL^{-1}$	Possible virulence factors	[141,142]
84	Tropolone	*Burkholderia plantarii*	*Penicillium oxalicum, Bacillus subtilis, Sarcina lutea, Saccharomyces pastorianus*			Iron-quelating property	[143,144,145]
	Cepalycin I and cepalycin II	*Burkholderia cepacia* JN106	*Saccharomyces cerevisiae, Cryptococcus neoformans, Candida albicans*		5-100 hemolytic units	Hemolytic activity due to their interaction with erythrocyte membrane cholesterol	[146]

VOC, volatile organic compound.
NRP, non-ribosomal peptide.
*Undetermined, however, the information was taken from other structurally related compounds.
